# 3D bioprinted scaffolds for osteochondral regeneration: advancements and applications

**DOI:** 10.1016/j.mtbio.2025.101834

**Published:** 2025-05-08

**Authors:** Jialin Lu, Yu Gao, Chen Cao, Hang Wang, Yaokuan Ruan, Keyi Qin, Hengyu Liu, Yanbo Wang, Pengju Yang, Yi Liu, Yingxue Ma, Zhifei Yu, Yinan Wang, Zhuan Zhong, Fei Chang

**Affiliations:** a Department of Foot and Ankle Surgery, The Second Hospital of Jilin University, Changchun, 130041, Jilin, China; b Department of Ophthalmology, The Second Hospital of Jilin University, Changchun, 130041, Jilin, China; c Department of Neurology, The First Hospital of Jilin University, Changchun, 130021, Jilin, China; d Department of Biobank, Division of Clinical Research, The First Hospital of Jilin University, Changchun, 130061, Jilin, China; e Key Laboratory of Organ Regeneration and Transplantation of the Ministry of Education, The First Hospital of Jilin University, Changchun, 130061, China

**Keywords:** 3D bioprinting, Osteochondral defect, Bioink, Gradient scaffold, Biomaterial, Tissue-forming cell, Signaling factor

## Abstract

Osteochondral defects, involving concurrent damage to articular cartilage and subchondral bone, pose significant clinical challenges due to their complex hierarchical structure and limited self-healing capacity. Traditional repair strategies often fail to replicate the biomechanical and biological gradients inherent to native osteochondral tissue, leading to suboptimal outcomes. Three-dimensional (3D) bioprinting has emerged as a transformative approach, enabling precise spatial deposition of biomaterials, cells, and signaling factors to construct biomimetic scaffolds with tailored gradients. This review systematically examines the physiological and pathological features of osteochondral units, emphasizing their zonal heterogeneity in extracellular matrix composition, mechanical properties, and cellular organization. Advancements in 3D bioprinting technologies are examined, and their efficacy in fabricating multi-layered and gradient scaffolds is evaluated. Key components of bioinks are discussed, focusing on optimizing bioink rheology, biocompatibility, and functional integration. Innovative strategies for embedding biochemical cues and designing continuous structural gradients are explored to address challenges in interfacial stress distribution and cell differentiation control. Furthermore, the design principles of biomimetic gradient scaffolds are highlighted for their critical role in facilitating osteochondral tissue regeneration. Finally, future directions are proposed, including high-resolution volumetric bioprinting, dynamic biomaterial development, and gene-activated scaffolds, aiming to bridge the gap between laboratory innovation and clinical application in osteochondral regeneration. This comprehensive analysis provides a roadmap for advancing 3D bioprinted solutions toward functional restoration of complex osteochondral defects.

## Graphical abstract

**Figure:**
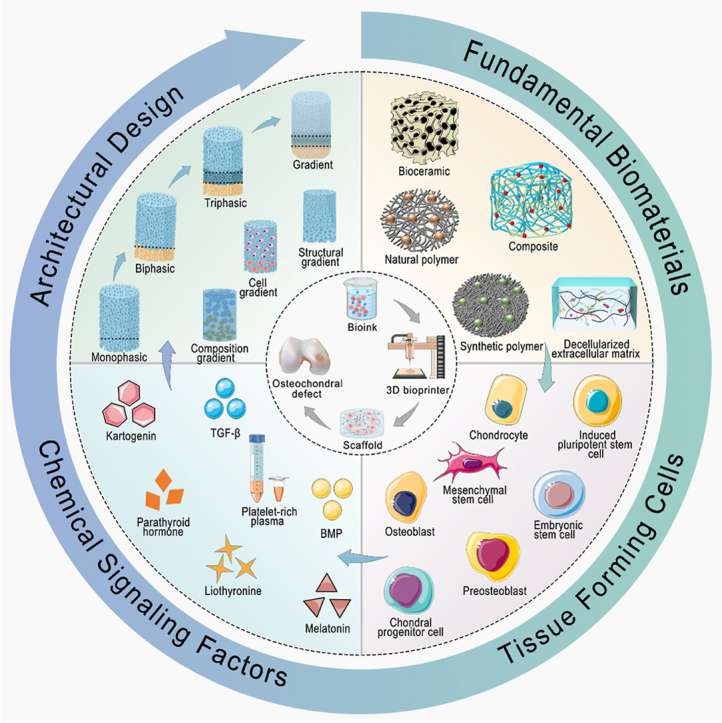

## 1 Introduction

Osteochondral defects refer to damage affecting both articular (hyaline) cartilage and the underlying subchondral bone, typically caused by trauma, joint diseases, or aging [1]. These defects predominantly occur in the knee and ankle joints but can also affect other areas, including the hands and spine [2]. Osteochondral lesions have the potential to progress to the surrounding cartilage and deeper subchondral bone, resulting in joint stiffness, pain, and functional impairment [1,3]. If left untreated, these conditions may ultimately lead to joint destruction and osteoarthritis. Osteochondral defects impose substantial physical and psychological burdens on individuals, as well as significant socioeconomic challenges worldwide [4].

Osteochondral tissues exhibit a complex, layered structure. The upper articular cartilage, the intermediate cartilage-bone interface, and the underlying subchondral bone collectively form an intact osteochondral unit, both structurally and functionally [2]. A natural gradient is present within these tissues, characterized by continuous variations in material composition, microstructure, and mechanical and biological properties from the cartilage surface to the subchondral bone [5]. Specifically, the density and alignment of various cell types, the content and orientation of extracellular matrix (ECM) fibers, the avascular and impermeable nature of cartilage, and the vascularization and porosity of subchondral bone all demonstrate gradient transitions [6]. Moreover, osteochondral tissues exhibit a gradient transition from the softer articular cartilage to the stiffer subchondral bone, which holds significant implications for osteochondral tissue engineering [5].

Various clinical methods are employed to treat osteochondral defects, such as palliative, reparative, and regenerative therapies. However, these treatments still face significant limitations and don't always lead to the best outcomes in repairing bone and cartilage [7,8]. To address these issues, osteochondral tissue engineering has been suggested as a way to develop more effective treatments [9]. Over the past few decades, osteochondral tissue engineering has rapidly developed and demonstrated significant potential as an effective solution for osteochondral defects. Despite the benefits of traditional osteochondral tissue-engineered scaffolds, such as cost-effectiveness and ease of use, they encounter several challenges. These include difficulties in customizing scaffold shapes for precise fit to the injury site, challenges in flexible design and precise control over the scaffold's microstructure, and the creation of gradient scaffolds.

To address these challenges, three-dimensional (3D) printing, particularly 3D bioprinting, has emerged as a promising solution. 3D bioprinting overcomes the limitation of only being able to add cells and growth factors to the scaffold surface at later stages by simultaneously co-printing biomaterials, tissue-forming cells, and signaling factors [10]. Moreover, this technology not only simulates the gradient structure and macroscopic defect morphology of bone and cartilage but also precisely controls micro-features, including the geometric shape, size, and porosity of pores [11]. As a result, 3D bioprinting has successfully overcome the technical challenges in traditional osteochondral tissue engineering and shows great potential in constructing complex, biomimetic osteochondral tissues.

**Fig. 1:**
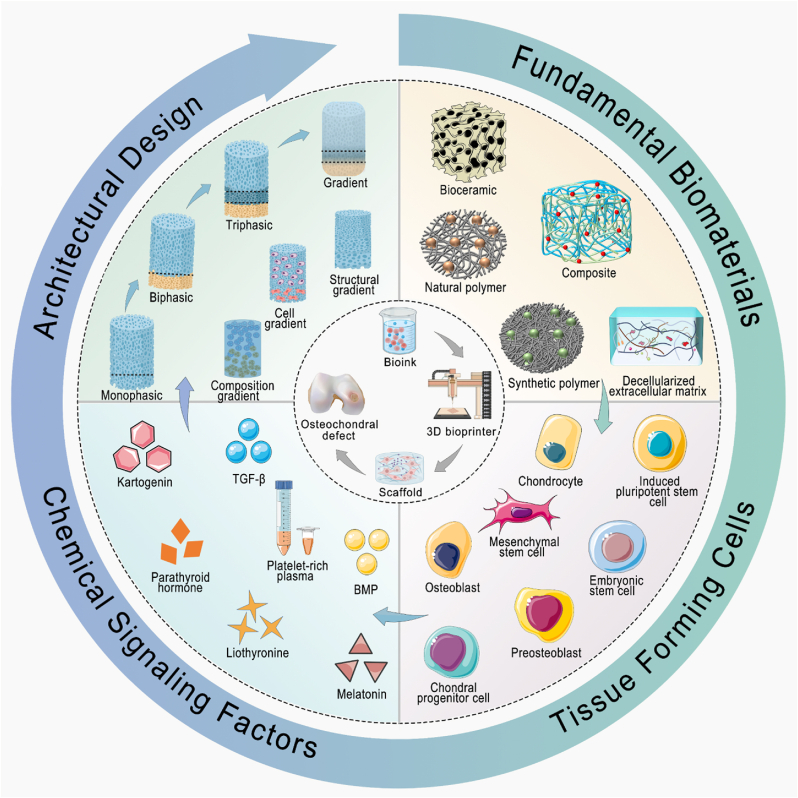
Schematic diagram of 3D bioprinting for osteochondral tissue engineering. BMP: bone morphogenetic protein; TGF-β: transforming growth factor-β.

This article reviews the advancements and applications of 3D bioprinting technology in the field of osteochondral regeneration. It begins by detailing the unique and complex physiological structure of osteochondral tissue, as well as the pathological changes associated with its injuries. Subsequently, the article summarizes the key elements of 3D bioprinting technology applied in osteochondral tissue engineering, including the formulation of bioinks, tissue-forming cells, and signaling factors for regulation. Additionally, this review particularly focuses on 3D bioprinted osteochondral composite scaffolds with layered and continuous gradient structures (Fig. 1).

## 2 Physiology and pathology of osteochondral tissue

### 2.1 Organizational structure of osteochondral unit

**Fig. 2:**
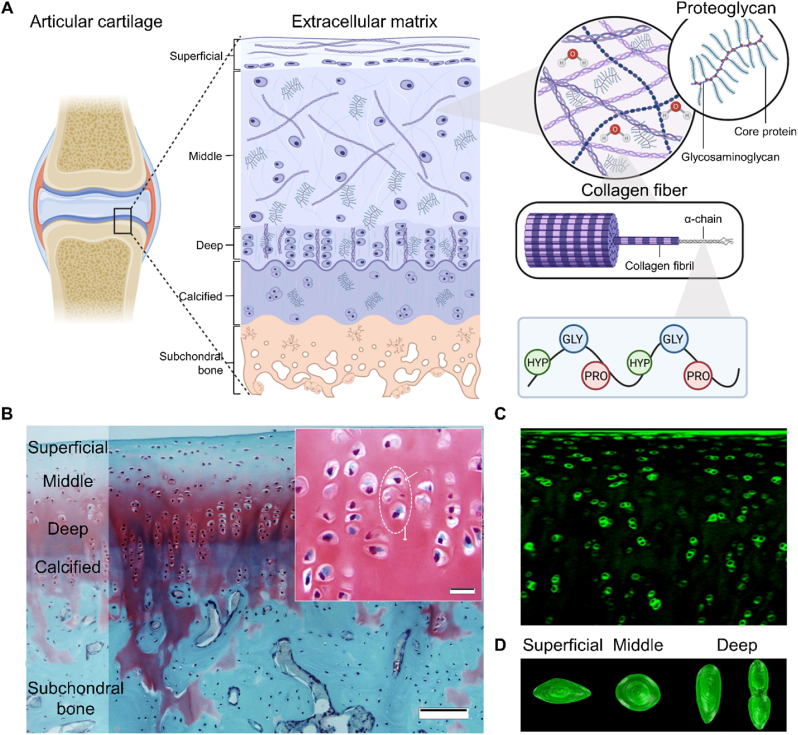
The schematic diagram and representative histological images of the osteochondral unit. (A) Graphical illustration of the zonal structure in the osteochondral unit. From top to bottom, they are superficial zone, middle zone, deep zone, calcified zone, and subchondral bone as depicted in the text. Within these zones, the content and architecture of extracellular matrix, as well as the morphology of chondrocytes exhibit a continuous gradient variation. Reproduced with permission [14]. 2023, Frontiers. (B) Safranin O/Fast Green staining identifies the zonal structure of healthy osteochondral tissue derived from a rabbit knee joint. Reproduced with permission [15]. 2018, Elsevier. (C) The fluorescence confocal image of an osteochondral tissue block immunolabeled for type VI collagen. (D) Typical shapes of chondrocytes in each zone under compression. Chondrocytes exhibite significant changes in height, shape, and volume. Reproduced with permission [16]. 2007, Elsevier. (For interpretation of the references to colour in this figure legend, the reader is referred to the Web version of this article.)

Effective design of biomimetic bioprinted osteochondral scaffolds requires a comprehensive understanding of the structural and functional characteristics of osteochondral units. An intact osteochondral unit consists of the upper articular cartilage, the intermediate cartilage-bone interface, and the underlying subchondral bone. The cartilaginous component is divided into four distinct zones: the superficial zone, middle zone, deep zone, and calcified zone [2]. The superficial, middle, and deep zones, collectively referred to as articular or noncalcified cartilage, constitute approximately 10–20 %, 40–60 %, and 30–40 % of the total articular cartilage thickness, respectively [12]. The tidemark demarcates the boundary between the noncalcified and calcified cartilage. Beneath the calcified cartilage lies the subchondral bone, with the interlocking structure formed between these two layers known as the cement line [13]. ‌Consequently, the osteochondral unit exhibits a unique gradient, characterized by continuous variations in material composition, microstructure, and mechanical and biological properties, spanning from the upper articular cartilage to the underlying subchondral bone (Fig. 2) [5].

#### 2.1.1 Articular cartilage

Articular cartilage, located on the surface of movable joints, is lubricated at the surface and functions as a cushion to reduce friction between adjacent bones. As a type of hyaline cartilage, articular cartilage is predominantly composed of ECM and chondrocytes. Unlike elastic cartilage and fibrocartilage, the ECM of articular cartilage primarily consists of water, type II collagen fibers, and proteoglycans [17]. In addition to type II collagen, articular cartilage contains smaller amounts of types V, VI, IX, X, and XI collagen [18]. The interactions among collagen molecules form a stable structural framework capable of withstanding external tension, imparting elasticity to the tissue. The diameter and orientation of collagen fibers vary across the cartilage zones [14,19]. In the superficial zone, the collagen fibers are the smallest in diameter, measuring approximately 30–35 nm. These fibers are organized in a highly parallel orientation relative to the joint surface, effectively distributing shear forces during joint movement. In the middle zone, the collagen fibers exhibit an increased diameter and are arranged randomly, allowing them to withstand stress from multiple directions. In the deep zone, the diameter of the collagen fibers increases further, reaching 40–80 nm. These fibers are oriented perpendicularly to the joint surface, thereby resisting compressive forces.

Proteoglycans are primarily composed of aggrecan aggregates, which consist of strands of hyaluronic acid (HA) and aggrecan monomers [20]. The molecular structure of proteoglycans is characterized by a high density of negatively charged hydroxyl and sulfate groups [21,22]. Additionally, the intrinsic network of proteoglycans contains numerous pores that facilitate the efficient hosting of cations and fluid molecules. Due to their negative charge and hydrophilic properties, proteoglycans resist fluid flow and can withstand compressive stress. Proteoglycan content is lower in the superficial zone and progressively increases with depth towards the deeper layers [12]. Concurrently, the fluid content within articular cartilage decreases with depth, ranging from 94.2 % in the superficial layer to 78.1 % in the deep layer. This change is attributed to the restricted expansion of proteoglycans imposed by the collagen fiber network in the deep zone. Given the positive correlation between water content and permeability, the deep zone exhibits very low permeability. As a result, fluid movement through this zone is negligible, and shear force at the osteochondral interface is maximized.

Chondrocytes, embedded within the ECM of articular cartilage, play a critical role in cartilage metabolism. They are responsible for the synthesis, organization, and maintenance of all components of the cartilage matrix. The morphology of chondrocytes is adapted to the mechanical stress in their microenvironment, exhibiting gradual variations across the different layers of articular cartilage [16]. In the superficial zone, chondrocytes are immature, exhibit an oblate shape, and are scattered singly across the cartilage surface. In the middle zone, chondrocytes adopt a rounded shape, undergo hypertrophy, and are randomly arranged. The deep zone is characterized by spherical chondrocytes surrounded by aligned collagen fibers.

#### 2.1.2 Osteochondral interface

The tidemark and calcified cartilage serve as an interface between the soft hyaline cartilage and the underlying subchondral bone. Located beneath the deep zone of articular cartilage, the tidemark appears histologically as a thin basophilic band that demarcates the noncalcified and calcified cartilage [9]. The collagen fibers across the tidemark are continuous with those in both the noncalcified and calcified cartilage, establishing a strong connection between the two. The tidemark exhibits a complex 3D structure. Its undulating shape creates a geometric pattern that helps resist shearing forces from a biomechanical perspective.

Calcified cartilage is located above the subchondral bone and is characterized by increased density and reduced levels of collagen fibers, water, and chondrocytes compared to articular cartilage [23]. The hypertrophic phenotype of chondrocytes and the process of calcification occur simultaneously in calcified cartilage. Mature cartilage cells secrete type X collagen, alkaline phosphatase (ALP), osteopontin, and matrix metalloproteinase (MMP)-13 [24].

Through endochondral ossification, calcified cartilage exhibits properties of both cartilage and bone. It provides structural integration between the flexible noncalcified cartilage and the rigid subchondral bone, facilitating the transfer and distribution of mechanical loads during joint motion [25]. Specifically, calcified cartilage buffers impact forces and accommodates variable shear stresses. Shear stresses from articular cartilage are converted into compressive stresses and transmitted to the subchondral bone via calcified cartilage [12]. These properties mitigate the impact forces exerted on the joint during movement, thereby reducing the risk of compression or tearing of articular cartilage. Biologically, calcified cartilage acts as a barrier, restricting material exchange between articular cartilage and subchondral bone. Furthermore, calcified cartilage prevents vascular invasion and the calcification of articular cartilage [26].

#### 2.1.3 Subchondral bone

The subchondral bone is the final region of the osteochondral unit and is separated from the calcified cartilage by the cement line. Structurally and physiologically, the subchondral bone can be divided into two regions: the subchondral bone plate (or subchondral cortical bone) and the subchondral trabecular bone (or subchondral cancellous bone) [2]. The subchondral bone plate is located in the upper layer, with a depth ranging from 10 μm to 3 mm across different body regions [27]. This plate is characterized by dense bone tissue, low porosity, and a sparse vascular network. The subchondral trabecular bone is located in the lower layer, with a thickness of approximately 6 mm and proximity to the bone marrow cavity. This region is characterized by a randomly arranged trabecular bone structure with high porosity, abundant blood vessels, and an active metabolic environment.

At the microscopic level, subchondral bone primarily consists of the ECM and a diverse array of cells. The organic components of the ECM are primarily collagen fibers, with type I collagen constituting approximately 90 % of the total collagen content [19]. The remaining amorphous interfibrillar matrix consists predominantly of proteoglycans and associated proteins, including osteonectin, osteopontin, and osteocalcin. These organic components provide elasticity and flexibility to subchondral bone. Inorganic components account for approximately 50 % of the dry weight of the ECM, with calcium hydroxyapatite (HAP) crystals being the most prevalent [28]. Other constituents include biological carbonates, citric acid, and ions such as magnesium, potassium, and sodium. Inorganic components collectively contribute to the rigidity of bone. Together, collagen fibers and inorganic minerals form a sophisticated hierarchical structure that contributes to the ultimate hardness and resistance of subchondral bone [29].

The cellular composition of subchondral bone includes osteoblasts, osteoclasts, osteocytes, and mesenchymal stem cells (MSCs). Osteoblasts synthesize HAP and contribute to new bone formation, while osteoclasts mediate bone resorption. Osteocytes regulate the interactions between osteoblasts and osteoclasts, maintaining bone homeostasis [19]. Additionally, subchondral bone is highly vascularized and contains numerous peripheral nerves. This network not only supplies essential nutrients to the osteochondral tissue but also facilitates timely responses to external stimuli. Therefore, compared to articular cartilage, subchondral bone confer a greater regenerative capacity. However, lesions with extensive defects often surpass the inherent self-healing capacity of subchondral bone and require appropriate treatment.

The primary function of subchondral bone is to support cartilage and transmit joint loads to the underlying bone [30]. The compact subchondral bone plate provides structural support, while the porous subchondral trabecular bone offers elasticity and absorbs impact during load transmission. The specific structure of subchondral bone minimizes and redistributes axial forces, cushions shocks through deformation and stress transmission, and prevents excessive stress-induced cartilage damage [31].

### 2.2 Mechanical properties of osteochondral unit

The structural and mechanical properties of osteochondral tissue vary from the articular cartilage surface to the subchondral bone. Structurally, porosity, permeability, and vascularization increase from articular cartilage to subchondral bone. Articular cartilage has a gel-like structure, with porosity ranging from 60 % to 85 % [19]. Its permeability is relatively low, with values of 9.313 x 10^−16^, 8.011 x 10^−16^, and 7.117 x 10^−16^ m^4^/N in the superficial, middle, and deep zone, respectively [32]. This low permeability is crucial for preventing the loss of synovial fluid and promoting nutrient transport to chondrocytes. The subchondral bone plate has a porosity of 5 %–30 % and greater stiffness, whereas the subchondral trabecular bone has a porosity of 30 %–90 % and lower stiffness [19]. The permeability of subchondral bone is measured at 2 x 10^−15^ m^4^/N and decreases with a reduction in osteocyte density [32].‌ Furthermore, the porous architecture of subchondral bone contains blood vessels and nerve fibers, which supply nutrients to osteocytes and facilitate metabolic waste removal.

In terms of biomechanical properties, the compression and elastic moduli increase, while the hydrostatic pressure and viscous modulus decrease from articular cartilage to subchondral bone [2]. This variation is primarily attributed to differences in material composition and structural organization between cartilage and bone. The compressive modulus and strength of articular cartilage exhibit a gradual increase from the superficial to the deeper layers, with the compressive modulus increasing from 0.2 MPa to 6.44 MPa, and the compressive strength rising from 0.005 MPa to 4 MPa [33]. Subchondral bone exhibits anisotropic properties due to the arrangement of its organic and inorganic components. In the subchondral bone plate, the transverse elastic modulus and longitudinal elastic modulus are 10.1 ± 2.4 GPa and 17.9 ± 3.9 GPa, respectively. The transverse tensile and compressive strengths are 53 ± 10.7 MPa and 131 ± 20.7 MPa, while the longitudinal tensile and compressive strengths are 135 ± 15.6 MPa and 205 ± 17.3 MPa [34]. On the contrary, trabecular bone performs better under compression than tension, with a compressive elastic modulus and strength ranging from 1 to 900 MPa and 1–10 MPa [35]. Osteochondral tissues exhibit a gradient transition from the softer articular cartilage to the stiffer subchondral bone, which holds significant implications for osteochondral tissue engineering.

In summary, the structure of the osteochondral tissue is highly complex, exhibiting significant heterogeneity across its regions. Current clinical repair strategies and traditional tissue engineering approaches struggle to replicate this intricate architecture. This limitation hinders the regeneration of osteochondral tissue with its highly organized arrangement of cells and ECM, thereby reducing the effectiveness of current treatment modalities. 3D bioprinting offers a promising solution by enabling the precise deposition of bioactive materials, cellular components, and signaling molecules. This technology holds substantial potential for reconstructing osteochondral tissues with a well-defined, layered architecture.

### 2.3 Pathology of osteochondral defects

Natural articular cartilage contains a limited number of cells and lacks blood vessels. As a result, it fails to undergo typical healing processes observed in other connective tissues, such as blood clot formation, inflammation, tissue necrosis, repair, and remodeling [17]. Furthermore, mature chondrocytes are incapable of producing sufficient ECM for effective tissue regeneration, significantly limiting the intrinsic healing capacity of articular cartilage. With aging, chondrocytes gradually undergo apoptosis, leading to a reduction in water content and proteoglycan levels within the cartilage matrix [36]. This decline renders articular cartilage particularly susceptible to damage. Additionally, conditions such as anti-inflammatory treatments, diabetes, and menopause have been shown to disrupt the cartilage structure and alter its stiffness, further increasing its vulnerability to degradation.

Compared to healthy osteochondral tissue, the cartilage in osteochondral defects undergo clonal proliferation in the early stages [37]. This process leads to cartilage degradation, thickening of the subchondral bone, and a reduction in trabecular volume. Immunohistochemical analysis reveals the degradation of the interterritorial matrix and alterations in the pericellular matrix of the cartilage. Besides, senescent chondrocytes are frequently observed in defective osteochondral tissue, indicating a strong association between chondrocyte senescence and osteochondral defects [38]. The aging of chondrocytes impairs the synthesis and secretion of type II collagen and proteoglycans, resulting in an imbalance between anabolic and catabolic ECM processes. Furthermore, senescent chondrocytes secrete factors associated with the senescence-associated secretory phenotype, which suppress ECM synthesis and activate matrix metalloproteinases [39,40]. This cascade of events accelerates cartilage degradation, ultimately contributing to the onset of osteoarthritis. Additionally, the absence of continuous collagen fibers bridging the calcified cartilage zones to the subchondral bone plate results in increased structural fragility at the bone-cartilage interface [31].

Although the degradation of articular cartilage has long been recognized as a hallmark of osteoarthritis, growing evidence highlights the critical role of subchondral bone in the progression and pathogenesis of osteochondral diseases. Recent studies have shown that microstructural and histopathological alterations in subchondral bone during the early stages of osteochondral disease occur before those in articular cartilage [41,42]. In healthy subchondral bone, a balance is maintained between bone resorption and deposition, enabling the tissue to dynamically adapt to mechanical loading. However, in osteochondral defects, this balance is disrupted, leading to increased thickness of the subchondral bone plate, alterations in the subchondral trabecular structure, formation of new bony structures at the joint edges, and the development of subchondral bone cysts [43]. Additionally, the highly innervated nature of the subchondral bone likely contributes to the joint pain experienced in the early stages of osteochondral disease. These changes in both articular cartilage and the underlying subchondral bone contribute to chronic pain and impaired joint function.

Osteochondral defects are classified into three distinct types based on lesion depth: (1) partial-thickness chondral defects, which are confined to the noncalcified cartilage and do not extend into the calcified cartilage; (2) full-thickness chondral defects, which involve the calcified cartilage; and (3) osteochondral defects, which entail complete damage to the full-thickness osteochondral structure, with exposure of the subchondral bone [44]. To accurately assess the severity of articular cartilage lesions for appropriate clinical management and research purposes, several classification systems have been developed. Among these, the Outerbridge classification is the most widely accepted [45,46].

**Fig. 3:**
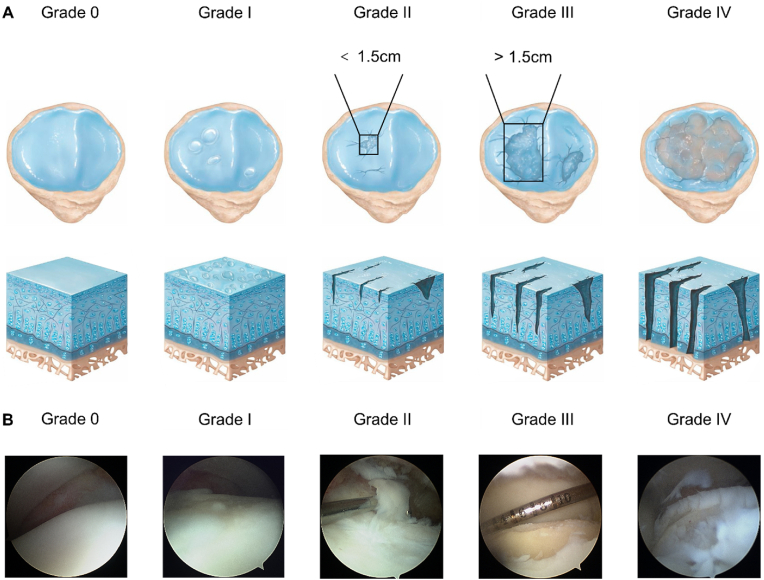
Schematic illustration and arthroscopic images of Outerbridge classification grades. (A) Schematic illustration of Outerbridge classification grades. Grade 0, normal cartilage. Grade I, focal areas of softening and swelling of cartilage. Grade II, partial-thickness chondral defect, with a defect diameter smaller than 1.5 cm. Grade III, full-thickness chondral defect, with a defect diameter larger than 1.5 cm. Grade IV, osteochondral defect, full-thickness chondral defect with exposure of the subchondral bone. Reproduced with permission [46]. 2020, Elsevier. (B) The intraoperative arthroscopic images of Outerbridge classification grades.

The Outerbridge classification consists of five progressive grades (Grade 0-IV), which assess the severity of chondral lesions and osteochondral defects (Fig. 3). Grade 0 corresponds to normal cartilage, Grade I indicates focal areas of cartilage softening and swelling, Grade II refers to partial-thickness chondral defects with a diameter smaller than 1.5 cm, Grade III denotes full-thickness chondral defects with a diameter larger than 1.5 cm, and Grade IV represents osteochondral defects characterized by full-thickness chondral defects with exposure of the subchondral bone. In addition to this classification, several other systems are available for more detailed lesion assessment, including the histological and histochemical grading system [47], the Osteoarthritis Research Society International (OARSI) Cartilage Histopathology Assessment System (OOCHAS) [48], and International Cartilage Repair Society (ICRS) Grading System [49].

## 3 Biomimetic 3D bioprinted scaffolds for osteochondral regeneration

Osteochondral scaffolds serve as temporary 3D structures for repairing osteochondral defects. They should mimic the native microenvironment while offering mechanical support to promote tissue repair and in situ regeneration. The natural osteochondral unit exhibits a distinct gradient, characterized by continuous variations in material composition, microstructure, mechanical and biological characteristics from the cartilage surface to the subchondral bone. To effectively mimic this gradient transition, the scaffold material, cell type, biological cues, scaffold structure, and mechanical properties must also exhibit a similar gradient [50]. Using computer-aided design, 3D bioprinting technology allows for the layer-by-layer deposition and stacking of materials to create 3D structures. This method offers several advantages, including high precision, rapid printing speed, the elimination of molds, and the ability to print personalized structures on demand [51].

**Fig. 4:**
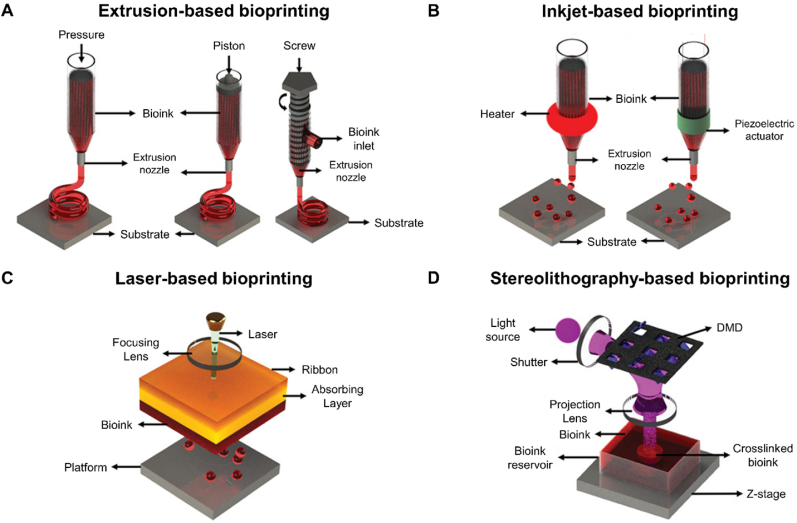
Schematic representations of the strategies currently used in 3D bioprinting for osteochondral tissue regeneration. (A) Extrusion-based bioprinting. From left to right: pressure-based, piston-based, and screw based strategies. (B) Inkjet-based bioprinting using (left) thermal or (right) piezoelectric actuation. (C) Laser-based bioprinting. (D) Stereolithography-based bioprinting. Reproduced with permission [54]. 2019, Wiley.

Unlike traditional 3D printing, 3D bioprinting employs biological substances as bioinks to fabricate tissue engineered constructs [52]. This innovative approach is designed to establish an optimal physiological microenvironment that supports cell survival, migration, proliferation, and differentiation. Moreover, 3D bioprinting enables the personalized fabrication of both macro- and microstructures of scaffolds on demand, surpassing the simple deposition of composite cells onto the surface of scaffold materials at later stages. By precisely controlling scaffold geometry, porosity, and pore topology, 3D bioprinting enhances cell-matrix interactions and coordinates mechanochemical signaling within the cellular niche. Consequently, it enables the construction of active and biologically functional tissues or organs [53]. Currently, 3D bioprinting technologies applied in osteochondral regeneration primarily include extrusion-based, inkjet-based, laser-based, stereolithography-based, and in situ bioprinting approaches (Fig. 4) (Table 1) [54].

**Table 1:** Comparative analysis of 3D bioprinting modalities across various dimensions.

	Extrusion-based	Inkjet-based	Laser-based	Stereolithography-based
Cell density	No limitation	Low, < 10^6^ cells/ml	Medium, < 10^8^ cells/ml	No limitation
Ink viscosity	Up to 6 × 10^7^ mPa/s	3.5–12 mPa/s	1–300 mPa/s	No limitation
Resolution	Medium	High	High	High
Print speed	Low	Fast	Medium	Fast
Cost	Medium	Low	High	Low
Advantages	Easy adapted for most materials	Rapid print speed, easy control, and wide applicability	High cell viability and high resolution	Rapid print speed, high resolution, and wide applicability
Disadvantages	High shear forces and low cell viability	Easy to clog	High cost and low applicability	Limited multimaterials

Since the advent of 3D bioprinting in osteochondral repair, scaffolds have been the subject of more in-depth and comprehensive research in areas such as biomaterials, cellular components, and signaling cues. This section summarizes recent advances in scaffolds from these perspectives.

### 3.1 Fundamental biomaterials for scaffold fabrication

Currently, 3D bioprinting technology remains in the developmental phase, with the limited availability of suitable biological inks serving as a significant barrier to its progress. Biomaterials serve as the primary components in bioink formulations, making the development of alternative biomaterials a critical research focus in the field [55,56]. Ideal biomaterials should possess the following properties: (1) Rheological properties, ensuring biomaterials are mechanically suitable for printing depending on the chosen 3D bioprinting technique; (2) High shape fidelity, referring to the ability of biomaterials to maintain structural integrity after deposition, as this influences printability directly; (3) Biocompatibility, meaning that biomaterials should be non-toxic and elicit minimal immune response when implanted in vivo; (4) Effective surface activity, capable of promoting cell differentiation and tissue regeneration while preserving cellular specific phenotype; (5) Adequate mechanical properties, capable of withstanding local stresses, such as tensile and compressive forces; (6) Good plasticity, enabling the design of a layered, continuous gradient scaffold structure with appropriate pore sizes and porosity. However, many biomaterials typically meet only a subset of these requirements, highlighting the need for novel biomaterials that satisfy all these criteria.

Scaffold compositions for osteochondral 3D bioprinting encompass natural and synthetic polymers, ECM-based materials, inorganic non-metallic substances, and composite materials derived from these components. This section provides a comprehensive review of recent advancements in 3D bioprinting materials for osteochondral defect repair. We examine their sources, properties, cross-linking mechanisms, and discuss their respective advantages and limitations in addressing osteochondral injuries.

#### 3.1.1 Natural polymers and their chemically modified derivatives

Natural polymers offer excellent biocompatibility, biodegradability, and low immunogenicity, making them widely used in 3D bioprinting [57]. The ability of natural polymers to incorporate bioactive motifs within their structure further enhances their applicability. Additionally, their derivatives allow for tunable biodegradability, mechanical properties, and specific biofunctions through chemical modifications of functional groups, ligands, and macromolecules [58]. Due to their biological origin, natural polymers and their derivatives are preferentially selected for the cartilage phase. In 3D bioprinting, natural polymers replicate the structure and function of the cartilage while providing signaling factors that enhance cell adhesion, growth, and proliferation [59]. Additionally, through various fabrication methods and the incorporation of components such as minerals, cells, and growth factors, natural polymers can be adapted for subchondral bone construction. However, challenges remain, including difficulties in purification, poor mechanical properties, uncontrollable degradation rates, and batch-dependent performance variability [9]. This section provides a comprehensive review of recent advancements in natural polymer-based biomaterials, including polysaccharides (e.g., alginate, HA, and cellulose), proteins (e.g., gelatin, silk fibroin, and collagen), and their derivatives in the development of 3D bioprinted osteochondral scaffolds (Table 2).

**Table 2:** Overview of commonly used natural polymers for osteochondral 3D bioprinting.

Polymer Type	Polymer Name	Crosslinking method	Stiff bone layer (Yes/No)	Advantages	Limitations	References
Polysaccharide-based biomaterials	Alginate	Physical (ionic interaction)	Yes	High functionality, biocompatibility, biodegradability, hydrophilicity, fast cross-linking, injectable for bioprinting, low cost	Poor mechanical strength, and stability, low cell-matrix interaction, difficulty in controlling pore parameters	[[60], [61], [62], [63], [64]]
	Hyaluronic acid	Chemical (Ultraviolet photo-polymerization)	No	Extracellular matrix component, biocompatibility, biodegradability, non-immunogenicity, easy to be functionalized	Inferior mechanical properties, rapid degradation, weak cell adhesion	[[65], [66], [67], [68]]
	Cellulose	Physical and chemical	Yes	Biocompatibility, low toxicity, well water retention, high mechanical strength	Weak solubility, limited standalone application	[[69], [70], [71]]
Protein-based biomaterials	Gelatin	Chemical	No	Biologically active for cellular interaction, low immunogenicity easy processing and functionalization	Poor mechanical properties, rapid degradation	[58,72,73]
	Silk fibroin	Physical (ionic interaction)	Yes	High mechanical strength, low immunogenicity, morphologic flexibility, well printability, high resolution	Source variability, low biodegradability, swelling capacity	[[74], [75], [76], [77]]
	Collagen	Physical and chemical	Yes	Extracellular matrix component, excellent cell-matrix interaction	Relatively low mechanical strength, fast biodegradability, potential of immunogenicity, risk of contamination	[65,[78], [79], [80]]

Alginate is a naturally occurring polysaccharide polymer, typically extracted from brown seaweed and certain bacteria [81]. It has been extensively studied and applied in the development of 3D bioprinted osteochondral scaffolds due to its biocompatibility, low toxicity, relatively low cost, and ability to undergo mild gelation upon the addition of divalent cations [60]. Alginate hydrogels effectively support the growth and proliferation of encapsulated chondrocytes, help maintain their phenotype, and enhance the expression of collagen type II and aggrecan [61,62]. Additionally, alginate hydrogels serve as a carrier for osteoblasts, osteoprogenitor cells, and MSCs in the context of bone regeneration [63]. However, alginate exhibits poor cell adhesion, weak cell interactions, and an uncontrollable degradation rate [64]. As a result, alginate is often chemically modified or combined with other polymers to improve its performance.

Physically crosslinked alginate hydrogels exhibit limited long-term stability and tend to lose their initial mechanical strength in physiological environments over a relatively short period [64]. This limitation can be addressed by incorporating additional biomaterials to form hybrid materials. Wu et al. combined alginate with β-tricalcium phosphate (TCP) to develop a bioprinted scaffold for bone regeneration and evaluated its physical and biological properties [82]. The results demonstrated that combining alginate's soft, cell-friendly matrix with the rigid, bone-mimicking structure of β-TCP produced a synergistic effect, promoting mechanical support, biodegradability, and osteogenic potential of the scaffold. Furthermore, this combination stimulated the proliferation of MG-63 cells and increased ALP activity within the tissues. Among the tested formulations, the 10 % alginate/β-TCP bioprinted scaffold exhibited optimal printability, swelling capacity, and degradation rate, facilitating superior cell viability and nutrient diffusion compared to the 12 % and 15 % formulations.

HA, a linear polysaccharide composed of 250 to 25,000 repeating disaccharide units, is a key component of aggrecan responsible for organizing the cartilage ECM into resilient structures [65]. HA performs a variety of functions, including cell lubrication, regulation of cell migration within a viscoelastic matrix, stabilization of reticular fibers, and protection of cells from mechanical stress-induced damage. Furthermore, HA interacts with various cell surface receptors and plays a pivotal role in critical cellular processes such as chondrocyte morphogenesis, proliferation, and the inflammatory response [58,66]. HA stimulates chondrocyte metabolism, leading to significant increases in collagen type II, aggrecan, chondroitin-6-sulfate, hydroxyproline, and DNA synthesis. Owing to these properties, HA has been widely employed as a printing material for the cartilage layer in 3D bioprinted osteochondral scaffolds. Researchers developed an HA-based cartilage layer scaffold loaded with chondrocytes using 3D bioprinting [83]. The results indicated that the survival and functionality of chondrocytes within the bioprinted structures were effectively maintained for up to 14 days in vitro.

Despite its bioactivity and excellent biocompatibility, HA lacks the physical properties necessary for bioprinting applications [67]. Specifically, HA solutions exhibit insufficient viscosity for reservoir stability during printing and lack the gelation ability essential for maintaining a stable 3D structure post-printing [68]. To overcome these limitations, researchers have exploited the carboxyl groups in HA molecules to react with substances containing hydroxyl or amino groups for chemical modification [65]. Among the various HA derivatives, tyramine-modified hyaluronic acid (HA-Tyr) has recently been introduced. A dual crosslinking process was employed to prepare the HA-Tyr bioink. The first step involved enzymatic crosslinking mediated by horseradish peroxidase and H2O2 to optimize extrusion and shape fidelity, followed by a second crosslinking step using green light in the presence of Eosin Y to stabilize the 3D construct [84]. Jahangir et al. used HA-Tyr hydrogel loaded with chondrocyte microspheres to fabricate the cartilage layer within an osteochondral scaffold via 3D bioprinting [84]. Live-dead staining of the embedded chondrocyte microspheres confirmed the biocompatibility of HA-Tyr. Reverse transcription (RT)--polymerase chain reaction (PCR) and histological staining further demonstrated that HA-Tyr promoted the expression of cartilage markers in chondrocytes and supported chondrogenic differentiation.

Additionally, HA can be further modified through methacrylation to produce methacrylated hyaluronic acid (HAMA), which enables photocrosslinking [85]. Under ultraviolet or blue light irradiation, HAMA undergoes crosslinking to form a stable 3D network structure. Abaci et al. developed an advanced embedded bioprinting platform utilizing photoactive HAMA hydrogels as a tunable bioink matrix for precise cell patterning [86]. MSC strands embedded within HAMA hydrogels exhibited significantly enhanced cellular spreading and increased cross-sectional aspect ratio when functionalized with arginine-glycine-aspartic acid (RGD) peptides. Moreover, HAMA serves as a hydrogel matrix or scaffold for subchondral bone regeneration through the incorporation of bioceramic particles and other osteogenic materials. For instance, HAMA doped with TCP particles significantly enhanced the osteogenic differentiation of MSCs. Furthermore, compared to higher-concentration formulations (10 % or 15 %), a lower-concentration formulation (5 %) more effectively promoted calcium deposition and ALP activity.

Cellulose is one of the most abundant natural polymers on Earth, found not only in plants but also in certain bacteria and algae. It is a linear polymer composed of D-glucose units linked by β-(1 → 4) glycosidic bonds, with a degree of polymerization reaching up to 18,000 and a molecular weight in the millions [69]. The rigidity of cellulose arises from hydrogen bond crosslinking between β-D-glucose molecules. As a natural polymer, cellulose has excellent biocompatibility, making it a valuable resource for bioink development in osteochondral 3D bioprinting. Currently, the primary forms of cellulose used bioinks include nanocellulose and cellulose derivatives. These materials serve either as main components of osteochondral scaffolds or as regulators facilitating their integration with other natural polymers.

Nanocellulose, derived from the degradation of lignocellulosic biomass, exhibits excellent mechanical properties, strong cell adhesion, good biocompatibility, and effective water retention due to its nanoscale dimensions [70]. Methylcellulose (MC), a cellulose methyl ether, is another important derivative. Due to its high biocompatibility, MC is commonly used in food and pharmaceutical applications [71]. Unlike cellulose, which is insoluble in water, MC hydrogels possess controlled solubility, enabling their wide application in tissue engineering and regenerative medicine. In osteochondral bioprinting, MC is used in three primary ways: as a support ink, as a sacrificial ink, and to enhance the viscosity of composite bioinks.

Gelatin is a collagen hydrolysate that contains numerous RGD sequences for cell adhesion and MMP target sequences for cell remodeling [72]. Unmodified gelatin has a gelation temperature that is unsuitable for in vitro cell culture or in vivo implantation, limiting its use in osteochondral tissue engineering [58]. Grafting methacryloyl groups onto gelatin is an effective modification strategy, rendering it chemically crosslinkable via photopolymerization. Gelatin methacryloyl (GelMA) demonstrates excellent biocompatibility, biodegradability, and a lack of immunogenicity. Moreover, GelMA facilitates easy regulation and micro-processing while preserving most of the functional amino acid sequences and cell adhesion properties inherent in gelatin [73]. As a result, GelMA has attracted growing attention as a novel biomaterial for applications in cell delivery and tissue engineering.

For instance, an extrusion-based 3D bioprinting strategy was developed to fabricate a GelMA-MSC scaffold, providing an appropriate microenvironment for cartilage repair [87]. The scaffold exhibited high biocompatibility and favorable physicochemical properties, further promoting the migration, proliferation, and chondrogenic differentiation of MSCs by upregulating microRNA-410. HAP exhibits chemical and structural similarities to natural apatite found in human bone mineral and is known for its biocompatibility, bioactivity, osteoconductivity, and osteoinductivity [88]. When combined with nano-HAP (nHAP), GelMA can be employed to develop a biomaterial for the repair of osteochondral defects. The incorporation of nHAP enhances the viscoelastic properties of GelMA and provides biological cues that help maintain the osteogenic phenotype of osteoblasts while promoting subchondral bone formation [84]. Furthermore, the researchers found that incorporating 10 % w/V β-TCP into GelMA hydrogel significantly promoted the mineralization of MSCs in a mixed osteogenic and chondrogenic medium [89].

Natural silk, derived from silkworm cocoons, consists of a silk fibroin protein core and a sericin protein coating. Silk fibroin possesses excellent biocompatibility, mechanical properties, a slow degradation rate, and abundant availability [74]. Fibroin hydrogels can be produced through various mechanisms that induce a conformational change in fibroin from an amorphous random coil to organized crystalline β-sheet structures [75]. Gelation methods for fibroin solutions include sonication, lyophilization, and treatments with acids, dehydrating agents, or ions [76].

In vitro study has indicated that silk fibroin hydrogels loaded with chondrocytes or MSCs support the production of cartilage-like ECM, including aggrecan and type II collagen. In addition, the compressive modulus of silk fibroin scaffolds reach 0.4 MPa, comparable to that of human articular cartilage [77]. These findings suggest that silk fibroin holds significant potential for promoting cartilage regeneration. However, due to its relatively low viscosity, frequent needle blockages, and slow gelation rate, the development of bioinks using silk fibroin alone remains challenging. Therefore, silk fibroin is often blended with other polymers to enhance its printability and overall functionality [90].

Collagen is the most abundant structural protein in the ECM. Type II collagen accounts for approximately 90 % of the dry weight of articular cartilage. The cross-linked network of type II collagen provides both structural integrity and mechanical strength to articular cartilage [18]. In contrast, type I collagen is the predominant ECM protein in bone tissue, comprising up to 90 % of its organic components and contributing significantly to its mechanical properties [91]. Collagen can be crosslinked through both physical and chemical methods. Physical crosslinking includes ultraviolet polymerization or dehydration heat treatment, while chemical crosslinking involves reactions with aldehydes, carbodiimides, or isocyanates [65].

Both in vivo and in vitro studies have demonstrated that hydrogel scaffolds constructed with type II collagen promote the proliferation and chondrogenic differentiation of MSCs [78,79]. A comparative analyse of type II collagen, alginate, and type I collagen hydrogels have highlighted differences in their regulatory effects on MSC chondrogenesis [78]. The results indicated that in the absence of growth factors, type II collagen hydrogels induced and maintained chondrogenic differentiation of MSCs, accompanied by upregulation of all cartilage-specific genes. On the other hand, type I collagen supports the growth, adhesion, and cartilage phenotype maintenance of chondrocytes and MSCs. Osteochondral scaffolds constructed with type I collagen hydrogels have demonstrated effective cartilaginous integration and cartilage formation up to one year post-implantation [92]. Furthermore, type I collagen has been shown to enhance osteoblast proliferation, migration, and phenotype maintenance, making it a valuable component for the bone layer in bioprinted scaffold [83]. However, the broader application of collagen in osteochondral tissue engineering is limited by its poor mechanical strength, suboptimal printability, and low post-printing shape fidelity [80]. To overcome these challenges, several strategies have been developed, including: (1) mechanical modification of bioink, (2) optimization of scaffold microstructure, and (3) enhancement of fabrication methods and techniques [93].

#### 3.1.2 Synthetic polymers

Although natural polymers exhibit excellent biocompatibility and support chondrogenic cell growth, proliferation, and phenotype maintenance. Their insufficient mechanical strength and uncontrolled degradation limit their widespread application in osteochondral tissue engineering. In contrast, synthetic polymers are produced through monomer polymerization, allowing for precise control over chemical structure and molecular composition. This enables the tailoring of their properties to meet specific requirements, offering tunable biocompatibility, biodegradability, mechanical strength, and biochemical characteristics [94]. Moreover, synthetic polymers provide greater consistency and controllability than natural polymers, making them well suited for large-scale industrial production [57]. However, synthetic polymers generally lack inherent biological properties, which can hinder cell adhesion. To address this limitation, surface bioactivity is often enhanced by incorporating tissue-forming cells or growth factors [95,96]. These modified materials are then employed in the construction of cartilage and bone layers within osteochondral scaffolds. Synthetic polymers used in the construction of 3D bioprinted osteochondral scaffolds include poly(ethylene glycol) (PEG), polylactic acid (PLA), poly(lactic acid-co-glycolic acid) (PLGA), and polycaprolactone (PCL) (Table 3).

**Table 3:** Overview of commonly used synthetic polymers for osteochondral 3D bioprinting.

Polymer Name	Crosslinking method	Stiff bone layer (Yes/No)	Advantages	Limitations	References
Poly(ethylene glycol)	Chemical (Ultraviolet photo-polymerization)	Yes	Versatility in processing and functionalization, good biocompatibility, low immunogenicity, mechanical adjustability	Biologically inert for cellular interaction, non-biodegradability, cell damage by ultraviolet radiation	[[97], [98], [99]]
Polylactic acid	Physical and chemical	Yes	Good biocompatibility, low toxicity, highly tailorability and printability	Release of acidic degradation products, inherent fragility	[100,101]
Poly(lactic acid-co-glycolic acid)	Physical and chemical	Yes	Good biocompatibility, highly tailorability in mechanical characteristics and biodegradability	Excessively high melting temperature, release of acidic degradation products	[57,102,103]
Polycaprolactone	Physical (temperature change)	Yes	Relatively low melting temperature, long-term mechanical stability, ease to manufacture, good biocompatibility	Poor bioactivity, hydrophobicity, slow degradation rate	[[104], [105], [106], [107], [108]]

PEG is a hydrophilic, biologically inert polymer widely used in various biomedical applications [97]. PEG-coated surfaces are frequently employed to regulate cell adhesion. In particular, photocrosslinked PEG hydrogels, such as poly(ethylene glycol) diacrylate (PEGDA), have been fabricated into scaffolds to support both in vitro cell growth and in vivo tissue regeneration [98]. PEGDA is a commonly used light-curing material due to its excellent biocompatibility, adjustable mechanical properties, and rapid curing speed. The mechanical and swelling properties of PEGDA hydrogels can be modulated by adjusting the monomer concentration, crosslinking density, and molecular weight of PEG. Furthermore, PEGDA hydrogels can be functionalized with bioactive molecules, such as growth factors, to enhance biological activity and enable controlled release and targeted delivery of therapeutic agents. In tissue engineering, PEGDA hydrogels serve as effective 3D bioprinting scaffold materials that provide an optimal growth environment for chondrocytes and osteoblasts. They promote cell proliferation and differentiation, while facilitating the repair and regeneration of cartilage and osteochondral tissues [99].

PLA is a linear polyester known for its thermostability, slow degradation rate, excellent biocompatibility, and low toxicity. The low viscosity and superior thermoplasticity make PLA well-suited for 3D bioprinting [100,101]. PLA-based bioinks have been proposed for fabricating various medical 3D bioprinting scaffolds aimed at facilitating the regeneration of bone, cartilage, and osteochondral tissues. PLA fiber scaffolds exhibit robust structural integrity and have been shown to support a significantly higher proliferation rate of seeded MSCs under physiological solutions [109]. In a rabbit model, MSC constructs seeded into PLA scaffolds were shown to form hyaline-like cartilage tissue. Additionally, Golebiowska et al. used biodegradable PLA to fabricate area or gradient scaffolds that provided mechanical strength and support osteochondral function [110]. The formation of multi-zonal and gradient scaffolds was confirmed using scanning electron microscopy imaging and micro-computed tomography scanning. Live/dead staining of the cell-laden hydrogel introduced into the cartilage zone revealed uniform cell distribution with high cell viability.

PLGA is a copolymer of lactic acid and glycolic acid. It is one of the most commonly selected synthetic polymers for constructing osteochondral scaffolds due to its biocompatibility and highly tunable properties, including mechanical characteristics and biodegradability [57]. PLGA scaffolds can be designed with varying pore geometries in the cartilage and bone layers, resulting in a bilayer scaffold that has demonstrated promising repair effects in rabbit models [102]. However, a key limitation of PLGA is its excessively high melting temperature (∼130 °C), which restricts the co-printing with live cells [103]. Another concern is the release of acidic degradation products, which may potentially induce inflammation.

PCL, a food and drug administration (FDA)-approved polyester, is commonly used in 3D bioprinting, favored for its cost-effectiveness, ease of modification, and favorable processing characteristics [104]. The semi-crystalline nature of PCL allows it to remain in a rubbery state at body temperature, offering superior toughness, elasticity, and mechanical strength [105]. Studies demonstrated that in a mature rabbit full-joint regeneration model, 3D printed PCL scaffolds successfully withstood the mechanical stresses associated with humeral condylar joint movement while maintaining adequate mechanical strength [106]. PCL-based scaffolds can be further customized in composition and internal geometry using various additive manufacturing techniques [107]. These findings suggest that implants reinforced with 3D bioprinted PCL can be designed to provide sufficient robustness for addressing large chondral and osteochondral defects. Daly et al. employed multimode composite 3D bioprinting techniques to fabricate PCL-based cartilage scaffolds [111]. These scaffolds not only enhanced the mechanical properties of cartilage to meet transplantation requirements but also incorporated micro-groove structures that provided an optimal environment for cellular growth.

Furthermore, PCL is an ideal material for fabricating highly porous scaffolds, which promote enhanced vascularization in the bone layer relative to the cartilage layer [108]. As a result, PCL is commonly employed in constructing the bone layer within osteochondral scaffolds. Additionally, the osteoinductive properties of bioceramics can be leveraged in combination with PCL to further enhance subchondral bone regeneration. Chen et al. used direct ink writing technology to fabricate PCL-HAP composite scaffolds for the bone layer, which supported the adhesion of human osteoblasts while maintaining their high activity levels [112]. This approach enhanced both the proliferation and mineralization of human osteoblasts over 21 days. Moreover, bioceramics can be incorporated into PCL polymers in the form of nanoparticles or nanowires to construct the bone layer with improved mechanical properties [113]. Notably, the degradation rate of PCL microfibers is relatively slow, typically requiring 2–3 years for complete degradation. Ideally, the degradation rate of PCL should be tailored to match the rate of tissue maturation, enabling long-term restoration of osteochondral tissue.

#### 3.1.3 Composites biomaterials

The polymeric biomaterials discussed above are highly versatile and compatible with most human or animal derived cells. However, bioinks made from single components are not always "jack-of-all-trades" because they may lack adequate mechanical properties or fail to fully support the proliferation and differentiation of target cells. To enhance the performance of bioinks or tailor them for specific applications, additional components are often integrated into the base materials, resulting in bioinks with improved properties. Composite, by combining the advantages of various polymers, offer a comprehensive set of benefits that a single polymer cannot provide. For example, they can deliver both biocompatibility and adjustable mechanical strength, making them highly attractive for osteochondral and cartilage tissue regeneration, thus achieving the synergistic effect of "1 + 1 > 2." Consequently, composite biomaterials made from two or more natural or synthetic polymers have garnered increasing attention in the fabrication of 3D bioprinted osteochondral scaffolds. The following section explores various composite biomaterials used in the construction of cartilage and subchondral bone layers, as well as full-thickness osteochondral scaffolds.

Alginate exhibits excellent biocompatibility but has relatively weak cell-material interactions [114]. While gelatin is not only biodegradable and biocompatible but also supports cell attachment and signaling functions [115]. The combination of alginate and gelatin enhances both the material properties and the biological interactions of the resulting biomaterial. For the cartilage layer, an alginate-gelatin hydrogel was bioprinted and integrated with 3D printed PCL scaffolds using fused deposition modeling to co-print a hybrid structure [112]. The bioprinted hybrid scaffold supported the encapsulation of chondrocytes at high density, maintaining 84 % cell viability and facilitating proliferation for up to 21 days. Additionally, Joshi et al. developed multifunctional silk-alginate-based bioinks capable of directing the differentiation of MSCs [116]. The researchers reported that the inclusion of silk enhanced the chondrogenic differentiation of MSCs, while the addition of phosphate groups to the bioink promoted osteogenic differentiation.

The low viscosity of alginate at cytocompatible concentrations limits its ability to achieve high shape fidelity during extrusion-based bioprinting. To address this challenge, Schütz et al. proposed the addition of MC as a thickener to temporarily increase viscosity during printing process [117]. The researchers found that a 3 % alginate and 9 % MC mixed hydrogel was suitable for accurately printing implants of clinically relevant sizes, while also encapsulating MSCs and ensuring their survival and metabolic activity. Furthermore, limitations associated with alginate, such as slow degradation, small pore size, and poor cellular migration and binding capacity, may hinder cellular uptake of signaling factors, particularly those complexed with nanoparticles larger than 10 nm [118]. To address this challenge, MC was proposed as a porogen in alginate-based bioinks. In vivo experiments have demonstrated that these pore-forming bioinks can accelerate the transfection of host or implanted cells [119].

Silk fibroin exhibits excellent biocompatibility and biodegradability and can be chemically modified via the side chains of specific amino acids to enhance biological activity and mechanical properties [120]. In a study by Deng et al., silk fibroin was grafted with parathyroid hormone (PTH) to form silk-PTH and covalently immobilized with methacrylic anhydride to create silk fibroin methacryloyl [121]. These modified forms of silk fibroin were then combined with gelatin methacryloyl (GM), which contains RGD sequences known to promote cell adhesion, to serve as bioinks for articular cartilage and subchondral bone regeneration. The addition of GM to the bioinks significantly enhanced their biological performance, making them suitable for 3D bioprinting applications [122]. Among the different bioink formulations, the GM + silk fibroin methacryloyl bioink exhibited favorable mechanical properties, whereas the GM + silk-PTH bioink inhibited chondrocyte hypertrophy, promoting the production of hyaline cartilage ECM [121].

The combination of HA with PLA offers suitable mechanical properties for use as a cell-carrier biomaterial in bioprinted constructs. This composite offers excellent printability, gelling ability, stiffness, and biodegradability [67]. The application of this HA-based bioink in 3D bioprinting facilitates the creation of biomimetic hybrid scaffolds for articular cartilage regeneration. HAMA is another widely used biomaterial due to its excellent biocompatibility and mechanical properties. However, standalone HAMA hydrogels lack temperature sensitivity. Despite this, the application of HAMA is restricted by poor mechanical properties. In contrast, GelMA is a temperature-sensitive material with specific amino acid sequences that promote cell adhesion [123]. Therefore, a HAMA/GelMA double-network hydrogel was developed, exhibiting excellent temperature sensitivity and mechanical properties comparable to native articular cartilage [113].

**Fig. 5:**
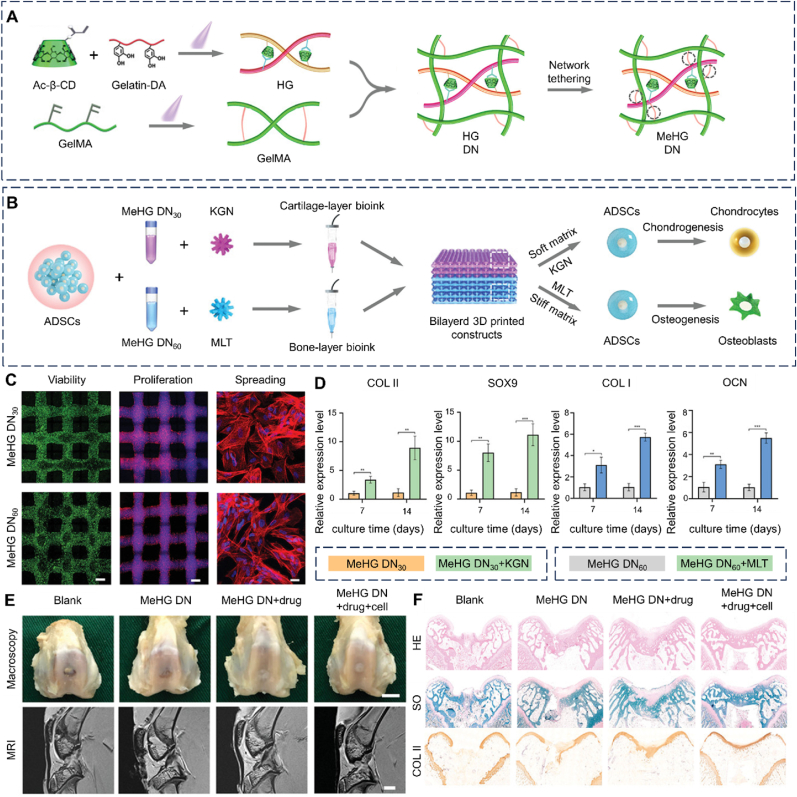
3D bioprinting of heterogeneous constructs providing tissue-specific microenvironment based on host-guest modulated dynamic hydrogel bioink for repairing osteochondral defects. (A) schematic illustration showing the fabrication process of network tethered host-guest modulated double-network hydrogel (MeHG DN). (B) Schematic illustration of 3D bioprinting biphasic ADSCs co-culture constructs with biomechanical and biochemical stimuli to create the tissue-specific microenvironment. (C) Cell viability, proliferation and spreading assay of ADSCs in 3D bioprinted constructs after cultured for 7 days. (D) Chondrogenic and osteogenic-related gene expression of ADSCs in each construct. (E) The gross and MRI images of the repaired osteochondral defects at 12 weeks post-surgery in different groups. (F) Histological images of H&E staining and SO staining, and immunohistological staining for type II collagen. Ac-β-CD: Acrylate β-cyclodextrin; GelMA: Gelatin methacryloyl; HG: Host-guest; HG DN: Host-guest and covalent double networks; MeHG DN: Methacrylated host-guest and covalent double networks; ADSCs: Adipose-derived mesenchymal stem cells; KGN: Kartogenin; MLT: Melatonin; MRI: Magnetic resonance imaging. Reproduced with permission [126]. 2022, Wiley.

Leveraging the biocompatibility of GelMA and the ease of operation of alginate methacryloyl (AlgMA), Zhang et al. investigated composite hydrogels of GelMA and AlgMA as customized bioinks for the fabrication of anisotropic bicellular living hydrogels [124]. The composite bioinks demonstrated shear-thinning behavior, temperature-responsive moduli, and photocrosslinking capabilities. These properties enabled direct room-temperature printing under cell-friendly extrusion pressures (≤70 kPa) and ensured structural stability after photocrosslinking [124,125]. In addition, a novel host-guest double-network hydrogel bioink, composed of dopamine-modified GelMA, GelMA, and acrylate β-cyclodextrin, was developed (Fig. 5) [126]. Acrylate β-cyclodextrin and dopamine side groups formed host-guest complexes as sacrificial bonds, conferring excellent toughness and resilience to the hydrogel, while the mechanical strength was enhanced through the polymerization of vinyl groups. This flexibility in polymeric network formation facilitated the fabrication of heterogeneous constructs with mechanical gradients. Additionally, acrylate β-cyclodextrin could encapsulate and store bioactive drugs, enabling their long-term sustained release.

**Fig. 6:**
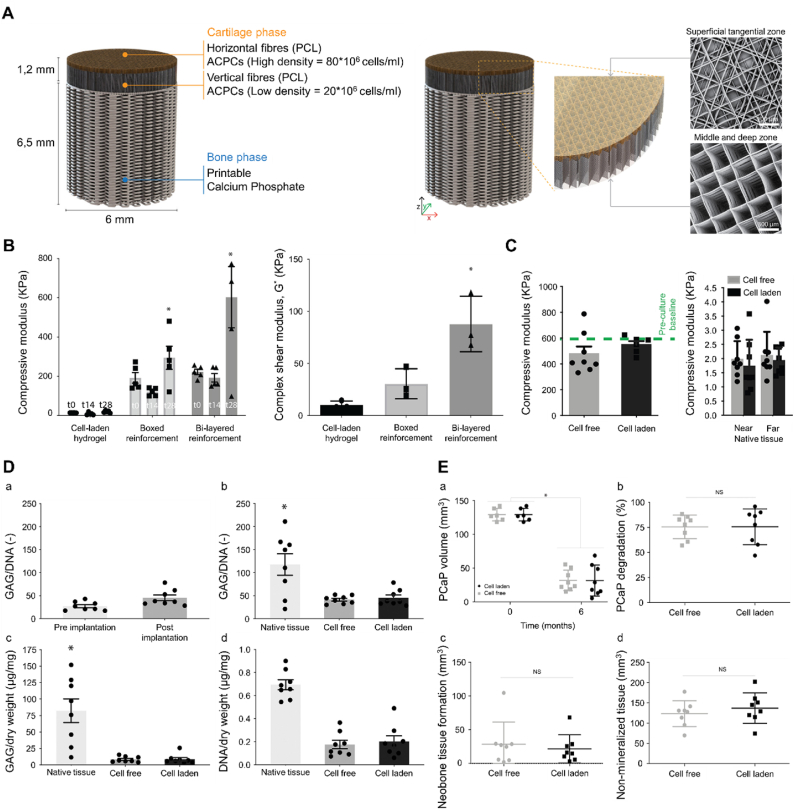
A study confirming the pivotal importance of structural reinforcement in bioprinted scaffolds. (A) Design details of the proposed multi-scale and multi-material osteochondral implant with details in the bilayered cartilage layer and regenerative pCaP bone phase. (B) Mechanical analysis of the osteochondral implants. (C) (Left) Compressive modulus of cell-free and cell-laden implants. (Right) Compressive modulus of native tissue measured near the defect site and far from the defect site. (D) Tissue quality of the cartilage layer of the implants after 6 months of implantation. (E) Quantification of micro-CT data after 6 months of implantation. PCL: Polycaprolactone; ACPCs: Articular cartilage-resident chondroprogenitor cells; GAG: Glycosaminoglycan; pCaP: Printable calcium phosphate; CT: Computed tomography. Reproduced with permission [127]. 2024, Wiley.

De Ruijter et al. reported the development of a converged bioprinted osteochondral implant composed of a GelMA-based cartilage layer reinforced with zonally arranged, melt-electrowritten PCL fibers (Fig. 6) [127]. This design provided long-term mechanical stability for neo-tissue in an orthotopic large animal model. The incorporation of highly organized (sub)micrometer-scale fibers significantly enhanced the compressive and shear properties of the hydrogel-thermoplastic composites, bringing them closer to the mechanical properties of native cartilage [128,129]. At the same time, the selection of relatively slowly degrading PCL as a structural material ensured the long-term retention of the implant's mechanical properties. Interestingly, the cell-free implants used as controls in this study exhibited abundant cell ingrowth and produced similar favorable results to those of the cell-containing implants. These findings underscore the hypothesis that mechanical stability plays a more critical role in the successful survival of the implant than the presence of cells and pre-cultured ECM.

#### 3.1.4 Decellularized extracellular matrix-based biomaterials

Despite significant advancements in polymeric biomaterials for 3D bioprinted osteochondral scaffolds, replicating the complex tissue microenvironment remains a challenge. To address this limitation, researchers have proposed the use of ECM-derived materials to fabricate biomimetic osteochondral scaffolds. Decellularized extracellular matrix (dECM), which refers to the ECM of tissues that has been isolated from its resident cells, has emerged as a promising natural biomaterial for tissue engineering [130]. dECM offers excellent biocompatibility, along with the safety and non-toxicity of both the material and its degradation products [131]. It not only provides a physical framework that supports tissue integrity but also creates a specific microenvironment conducive to the survival, proliferation, and differentiation of resident cells.

For cartilage regeneration, decellularized cartilage extracellular matrix (DCM) is considered a superior biomaterial compared to synthetic ECM. DCM contains various bioactive components, including collagen type II, glycosaminoglycans, and specific growth factors and cytokines, all of which are essential for guiding cartilage formation [132]. To minimize adverse immunological responses, nucleic acids and other cellular components are removed, while most ECM components, such as type II collagen and Sox-9, are preserved [133]. Similarly, decellularized bone extracellular matrix (DBM) has been explored as an ideal biomaterial for bone regeneration. Its native 3D architecture and resemblance to bone matrix, particularly the osteoinductive and biomechanical properties, make it highly suitable for bone tissue engineering [134]. Thus, dECM-based bioprinted scaffolds are effective in promoting stem cell migration, proliferation, and differentiation, thereby enhancing osteochondral repair following implantation.

Ryu et al. investigated the potential of a 3D bioprinted matrix composed of lyophilized costal-derived cartilage matrix and minimally manipulated adipose ECM to repair a critical-size femoral cartilage defect [135]. The experimental group demonstrated higher Magnetic Resonance Observation of Cartilage Repair Tissue(MOCART) scores and more organized cartilage repair compared to other groups. These findings suggest that the combination of cartilage powder and adipose tissue repairs cartilage in a manner resembling native tissue. In addition, Wu et al. developed a clinically translatable strategy for preparing heterogeneous DCM [136]. They obtained a DCM-based mixed bioink with good biocompatibility and printability, which expressed high levels of cartilage-related growth factors. Using 3D bioprinting, this bioink was uniformly printed with adipose-derived mesenchymal stem cells (ADMSCs) isolated from the infrapatellar fat pad (IPFP) to create biomimetic scaffolds. ADMSCs encapsulated in the scaffolds exhibited excellent proliferation and differentiation capabilities, with a strong potential for chondrogenic differentiation. Besides, no severe immunogenic response was detected following subcutaneous implantation, which was primarily attributed to the natural endogenous components of DCM.

The mechanical strength of dECM is often compromised due to the loss of native structure during the homogenization and solubilization processes [137]. Silk fibroin, renowned for its excellent mechanical properties, biocompatibility, and biodegradability, has been widely studied in 3D bioprinting applications. A bioink composed of dECM and silk fibroin was developed to fabricate bilayered scaffolds that mimic natural osteochondral tissue, with precise control over composition, mechanical properties, and growth factor release in each scaffold layer [138]. The results showed that each scaffold layer maintained appropriate mechanical strength and degradation rates while releasing encapsulated growth factors in a controlled manner, thus promoting the directional differentiation of bone marrow-derived mesenchymal stem cells (BMSCs). Moreover, researchers combined the benefits of dECM with photocrosslinked PEG to fabricate a bioprinted osteochondral construct that solidifies rapidly upon exposure to visible light [97]. This construct offers two main advantages for osteochondral repair. On one hand, it preserves the cartilage-inducing properties of natural cartilage ECM, thereby supporting chondrogenic differentiation of MSCs. On the other hand, the incorporation of PEGDA increases the hydrogel's porosity, enhancing its compressive modulus and providing greater versatility and flexibility in the fabrication process.

In summary, dECM provides a microenvironment that closely mimics native osteochondral tissue, supporting cell migration, proliferation, and differentiation. Cells encapsulated in DCM or DBM hydrogels recognize and interact with the surrounding matrix, enhancing chondrogenic or osteogenic differentiation and promoting tissue maturation. However, the lack of standardized decellularization protocols and limited reproducibility remain significant challenges that must be addressed in future research. Moreover, the decellularization process compromises the mechanical and biochemical integrity of the osteochondral tissue, further limiting its application [139].

#### 3.1.5 Bioceramics

Osteochondral defects involve both articular cartilage damage and subchondral bone injury. Therefore, selecting biomaterials that can support subchondral bone regeneration and mimic the structure and function of native subchondral bone is crucial for the design of biomimetic scaffolds. Natural polymers, synthetic polymers, and dECM primarily mimic the physiological environment and mechanical properties of articular cartilage. However, their limited mechanical properties and low structural fidelity render them insufficient for subchondral bone regeneration. Bioceramics are widely recognized for their excellent biocompatibility, surface bioactivity, and mechanical properties, making them essential components for subchondral bone reconstruction [140]. Research has demonstrated that bioceramics possess a composition similar to that of native subchondral bone and exhibit osteoconductive and osteoinductive properties, which promote new bone formation and enhance scaffold integration with surrounding tissues [141]. Additionally, their high compressive strength and low ductility provide structural stability and resistance to deformation. However, brittleness remains a major challenge to be overcome in the application of bioceramics [142]. Bioceramics include a range of inorganic biomaterials, such as HAP, TCP, silicate, and amorphous bioactive glass.

HAP is a calcium phosphate mineral abundantly present in the inorganic matrix of bone. It can be used in various forms to construct bioprinted osteochondral scaffolds, including bulk scaffold layers, nHAP particles, and nHAP-containing microspheres [143]. HAP increases local calcium ion concentrations, stimulates osteoblast proliferation, and promotes the growth and osteogenic differentiation of MSCs, thereby facilitating new bone formation [144]. Furthermore, its osteoconductive and osteoinductive properties contribute to the restoration of subchondral bone in osteochondral injuries. Multiphasic scaffolds containing HAP in the bone layer show enhanced integration with host tissue and newly formed bone, as well as improved vascularization [145]. Moreover, in bioprinted osteochondral scaffolds, HAP can enhance the mechanical properties of polymer materials, such as alginate and gelatin, bringing them closer to the properties of native bone [146]. However, a limitation of HAP is its low biodegradability, as studies have shown very limited or slow degradation in both small and large animal models [147].

TCP is another commonly used calcium phosphate material, notable for its lower formation temperature and its chemical composition, structure, and mechanical and biological properties that closely resemble those of natural bone mineral [148]. TCP exhibits excellent biocompatibility and osteoconductive properties and can self-harden through hydrolysis, forming a 3D structure that mimics the inorganic components of natural bone [149]. These properties make TCP a promising material for manufacturing customized scaffolds. A bioceramic capable of setting at physiological temperatures was developed using TCP, nHAP, and a custom-synthesized, biodegradable, and crosslinkable poloxamer for subchondral bone support [150]. This composite demonstrated a mild setting reaction at physiological temperature, enabling direct printing onto melt-electrowritten PCL meshes while preserving their microarchitecture. Besides, the bioprinted implant designed by de Ruijter et al. consisted of a 3D printed TCP-based bone phase, integrated with the cartilage layer through embedded PCL fibers generated by melt electrowriting to securely connect the cartilage and bone components of the osteochondral unit [127]. Six months after implantation, the bone compartment of the osteoconductive ceramic-based implant showed significant cellular infiltration and complete formation of new bone tissue.

Silicate-based bioceramics are bioactive materials composed of silicates with specific phase structures. These materials are biodegradable, with degradation rates largely determined by their chemical composition and structural characteristics. Over 20 types of silicate-based bioceramics have been synthesized through sol-gel, precipitation, and solid-state reaction methods. Most of these materials induce the mineralization of bone-like HAP in simulated body fluids, facilitating osteogenic bonding with bone tissue. Building upon these findings, the active mechanism of silicate bioactive ceramics has been extensively studied. Research has shown that these materials release various bioactive ions, particularly silicon ions, which significantly promote the osteogenic differentiation of stem cells and induce angiogenesis [151]. These properties make silicate-based bioceramics promising for bone tissue repair and soft tissue regeneration. Current studies suggest that silicon ions exert their osteoinductive effects primarily through the activation of the AMPK/ERK1/2, PI3K/Akt, and Wnt signaling pathways [152]. Consequently, the application of silicates has advanced the development of bioceramics from traditional calcium phosphate-based systems to calcium silicate-based systems with "active osteogenesis" capabilities.

**Fig. 7:**
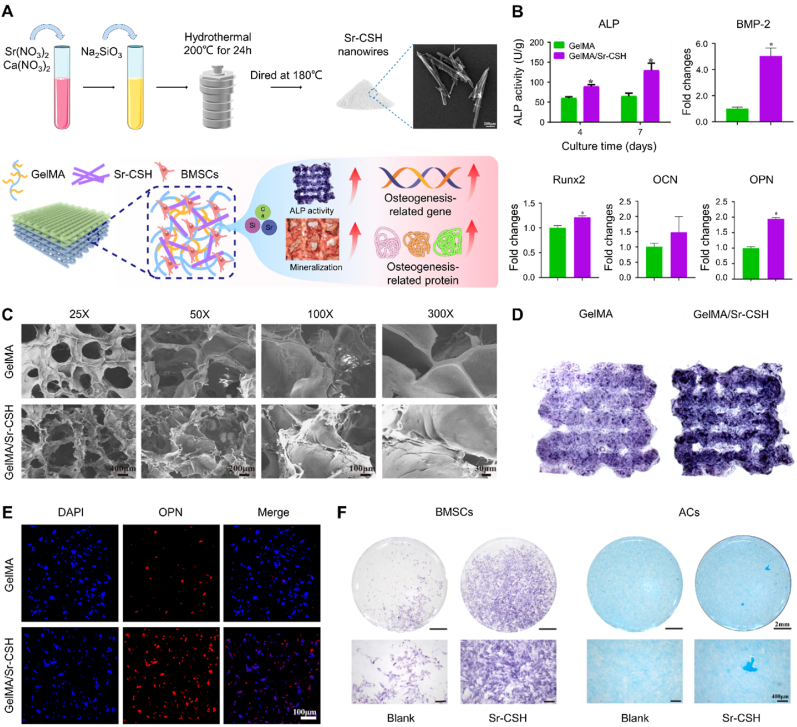
3D bioprinted biphasic multicellular living scaffolds facilitating the regeneration of osteochondral defects. (A) Schematic illustrating the synthesis of Sr-CSH nanowires and the osteogenic differentiation of BMSCs induced by GelMA/Sr-CSH. (B) Semiquantitative assessment of ALP activity and quantitative PCR analysis of osteogenesis-related genes in BMSCs within GelMA and GelMA/Sr-CSH 3D bioprinted scaffolds. (C) Scanning electron microscopy images of GelMA and GelMA/Sr-CSH based bioprinted scaffolds. (D) ALP staining images of GelMA and GelMA/Sr-CSH 3D bioprinted scaffolds. (E) Immunofluorescence staining of osteogenesis-related protein in GelMA and GelMA/Sr-CSH 3D bioprinted scaffolds. (F) (Left) ALP staining images of BMSCs cultured with the medium containing the ideal dilution of Sr-CSH extracts. (Right) Alcian blue staining images of ACs cultured with the medium containing the ideal dilution of Sr-CSH extracts. Sr-CSH: Strontium-substituted xonotlite; GelMA: Gelatin methacryloyl; ALP: Alkaline phosphatase; BMSCs: Bone marrow-derived mesenchymal stem cells; ACs: Articular chondrocytes. Reproduced with permission [154]. 2024, Wiley. (For interpretation of the references to colour in this figure legend, the reader is referred to the Web version of this article.)

A Li-Mg-Si bioceramic-containing bioink was successfully developed and applied to fabricate a multicellular scaffold mimicing osteochondral tissues using 3D bioprinting [153]. This bioink not only replicated bone components but also released bioactive ions that induced MSC-specific differentiation, promoting the concurrent regeneration of both cartilage and subchondral bone. As a result, the 3D bioprinted co-culture scaffolds exhibited excellent cellular activity, with the ability to simultaneously modulate the differentiation of multiple cell types in vitro and significantly accelerate the repair of large osteochondral defects in vivo. Furthermore, Yu et al. employed 3D bioprinting to construct a biphasic multicellular scaffold mimicing the native osteochondral tissue (Fig. 7) [154]. The cartilage layer was composed of GelMA hydrogel loaded with chondrocytes and BMSCs, while the subchondral bone layer consisted of strontium-substituted xonotlite (Sr-CSH)-modified GelMA loaded with BMSCs. The GelMA/Sr-CSH bioink degraded gradually and released bioactive ions, maintaining a microenvironment conducive to osteogenic differentiation. This environment stimulated BMSCs to express key osteogenic markers, including osteopontin, osteocalcin, and bone morphogenetic protein (BMP)-2.

Bioactive glass is an amorphous mineral that has gained increasing attention as a bone-phase material for osteochondral scaffolds. It contains network modifiers that trigger a cascade of events, leading to the formation of a bioactive HAP-like surface layer. This enhances bone cell adhesion, stimulates new bone formation, and supports the repair of surrounding soft tissues [155]. Bioactive glass-based osteochondral scaffolds also exhibit excellent mechanical properties and allow for the regulation of the degradation rate, significantly improving subchondral bone vascularization and increasing the compressive modulus [156,157]. Upon implantation in osteochondral defects, these scaffolds effectively reconstruct osteochondral tissue with hierarchical porosity.

#### 3.1.6 Metallic materials

Metallic materials exhibit mechanical properties similar to bone, allowing them to withstand high stress without deformation. This makes them ideal for constructing the subchondral bone layer in osteochondral scaffolds, where they fill defective bone and provide structural support, thus aiding in the repair of both bone and cartilage. The most commonly used metallic materials in biomedical implants include titanium and its alloys, tantalum, cobalt-chromium alloys, and stainless steel. Among these, titanium and its alloys are preferred for their superior biocompatibility, corrosion resistance, and mechanical strength. The formation of a dense titanium dioxide layer on the surface prevents corrosion and enhances integration with surrounding tissues. As to itanium alloy, Ti_6_Al_4_V is the most widely used due to its optimal balance of strength and ductility [158].

Based on the hypothesis that a rigid subchondral bone chamber promotes bone and cartilage regeneration, Yang et al. designed a biphasic osteochondral scaffold [159]. The subchondral bone chamber was constructed from a 3D printed titanium alloy network with large pores, providing robust and stable mechanical support for both bone and cartilage. The cartilage layer was composed of a freeze-dried collagen sponge with a microporous structure, facilitating chondrocyte adhesion and phenotype maintenance. PLGA infiltration further enhanced the mechanical properties of the cartilage chamber, providing a stable platform for cartilage regeneration. All materials used are FDA-approved for clinical applications, and in vitro tests confirmed their biocompatibility. Additionally, in vivo studies demonstrated that the biphasic scaffold, incorporating the titanium alloy layer, outperformed the monophasic scaffold in promoting bone and cartilage regeneration.

Similarly, Ouyang and colleagues proposed an innovative "living joint" strategy for repairing large osteochondral defects resulting from tumor resection or severe trauma [160]. This approach combined a custom 3D printed titanium alloy prosthesis with a silk fibroin hydrogel containing BMP-2 and transforming growth factor (TGF)-β3. In a rabbit osteochondral defect model, the living joint achieved weight-bearing functionality and exhibited both macroscopic and microscopic characteristics comparable to those of natural joints two months post-surgery. Additionally, the living joint significantly enhanced bone integration, resulting in a threefold increase in bone ingrowth and mechanical pull-out strength. Furthermore, it restored articular cartilage function, enabling the rabbits to walk and bear weight normally. This achievement marks a significant advancement in the design of bioactive bone prostheses and offers new hope for the treatment of large osteochondral defects.

However, the difference in elastic modulus between metal implants and bone tissue results in stress shielding, which leads to implant loosening and failure. Additionally, metal implants have several other limitations, including poor degradation, inadequate integration with surrounding tissues, and an inability to promote tissue regeneration. More critically, conventional 3D printing techniques for metals, such as selective laser melting and electron beam melting, require post-processing steps like heat treatment. These extreme conditions hinder the incorporation of cells and biochemical factors, rendering them unsuitable for bioprinting active osteochondral tissues. To address these challenges, researchers have explored metallic materials such as cobalt, zinc, strontium, and lithium, combined with polymers or bioceramics, to develop advanced bioinks. These bioinks enable the fabrication of active osteochondral scaffolds through mild printing processes, resulting in scaffolds with excellent mechanical properties and the ability to release metal ions, further promoting bone and cartilage regeneration.

Studies have shown that functional modification of materials with cobalt results in oxygen-scavenging materials with catalytic sites [161]. In addition, cobalt stimulates cells to express anti-inflammatory genes and promotes osteogenesis and angiogenesis [162]. Based on these principles, Shu et al. synthesized cobalt-doped chlorophosphate apatite (Co-ClAP) bioceramics with antioxidant properties and bioactivity using the molten salt method [163]. The researchers then printed porous Co-ClAP/PLGA scaffolds, using PLGA as a binder. These scaffolds promoted the adhesion, proliferation, and directed differentiation of chondrocytes and BMSCs under oxidative conditions by scavenging reactive oxygen species (ROS). It also downregulated the expression of inflammatory cytokines in immune cells, which created an anti-inflammatory microenvironment to support tissue formation.

Zinc possesses antioxidant and anti-inflammatory properties, exerting its effects by modulating the generation of free radicals in the body [164]. Its insulin-like properties also promote cartilage formation [165]. Studies have shown that zinc-cobalt bimetallic organic frameworks (Zn/Co-MOF) exhibit excellent ROS catalytic activity, comparable to that of peroxidase, catalase, and superoxide dismutase [166]. For instance, Shu et al. developed functionalized osteochondral scaffolds through in situ deposition of Zn/Co-MOF onto 3D bioprinted β-TCP scaffolds [167]. By adjusting the concentration of the Zn/Co-MOF solution, the researchers controlled the scaffolds' ability to scavenge ROS and their cellular compatibility. These functionalized scaffolds promoted osteogenic differentiation of rat BMSCs and maturation of chondrocytes, while simultaneously protecting them from oxidative stress and creating an anti-inflammatory environment. In vivo studies further confirmed the beneficial effects of the MOF-TCP scaffolds on osteochondral tissue regeneration.

Strontium is widely used in bone regeneration therapies. It has also been shown to induce macrophage M2 polarization, enhance the differentiation of human BMSCs into chondrocytes, and promote chondrocyte proliferation and maturation [168]. Specifically, lower concentrations (3-46 × 10^−6^ M) of strontium effectively promote cartilage repair, while higher doses (100-2000 × 10^−6^ M) are required for bone repair [169]. Mechanistic studies indicate that strontium enhances tissue regeneration by modulating both classic and non-classic Wnt signaling pathways, the HIF signaling pathway, and the Notch signaling pathway during osteogenesis and chondrogenesis [170,171]. Li et al. designed a 3D printed scaffold incorporating strontium and doped bioactive nanoglass for the repair of osteochondral defects [171]. The scaffold featured a precisely engineered pore structure and accelerated the recruitment, adhesion, and proliferation of chondrocytes and BMSCs through the sustained release of strontium, silicon, and calcium ions. In addition, silicon ions promoted osteogenic differentiation of BMSCs, macrophage M2 polarization, and angiogenesis in subchondral bone.

Lithium has shown promising potential in the treatment of osteochondral defects. Studies have demonstrated that lithium stimulates subchondral bone formation and increases bone mass in mice by activating the Wnt signaling pathway [172]. Besides, lithium selectively inhibits phosphorylation reactions, thereby protecting cartilage from degradation [173]. It stimulates chondrocyte proliferation and regulates the primary cilia in chondrocytes as well [174]. Building on these findings, Chen et al. synthesized high-purity lithium-calcium silicate (L_2_C_4_S_4_) powder using a sol-gel method and employed 3D printing to fabricate scaffolds with a uniform macroporous structure [175]. By adjusting the pore size (170–400 μm) and porosity (37–61 %), the compressive strength of the scaffold ranged from 15 to 40 MPa, surpassing that of conventional bioceramic and polymer scaffolds. Moreover, the 3D printed scaffold exhibited unique apatite mineralization capabilities and effectively promoted the adhesion, proliferation, and differentiation of both chondrocytes and human BMSCs.

In summary, metallic materials, with mechanical properties similar to bone tissue, are ideal for the subchondral bone layer in osteochondral scaffolds. Titanium, titanium alloys, tantalum, and cobalt-chromium alloys are widely used for their biocompatibility and corrosion resistance. However, metallic materials cause lead to stress shielding, and traditional printing techniques for metal face challenges in incorporating bioactive components. To address these challenges, current research focuses on developing novel bioinks that integrate metal ions, such as cobalt, zinc, strontium, and lithium, into biomaterials. These bioinks leverage antioxidant, anti-inflammatory, and signaling modulation properties, combined with mild printing processes, to create multifunctional scaffolds. By balancing mechanical performance with bioactivity, these innovations offer new opportunities for osteochondral repair.

### 3.2 Cellular components for 3D bioprinting osteochondral scaffolds

Tissue engineering and cell therapy have been integrated to enhance osteochondral defect repair. Cell-based therapies, including autologous chondrocyte implantation and matrix-associated autologous chondrocyte implantation, are widely recognized clinical strategies for treating osteochondral defects. However, directly implanting a chondrocyte graft into focal osteochondral defects with complex geometries remains a challenge for orthopedic surgeons. To address this, 3D bioprinting has been proposed as a solution, enabling the fabrication of scaffolds embedded with tissue-forming cells. Cells within the scaffolds are capable of chondrogenic and/or osteogenic differentiation, creating engineered osteochondral units. Bioinks containing living cells influence the interaction between the scaffold and the surrounding osteochondral tissue, ultimately influencing the overall healing process. Although a variety of cellular components have been used in engineered scaffolds, the types of cells currently employed in 3D bioprinting for osteochondral defect repair remain limited. These include host tissue-derived cells, such as chondrocytes, cartilage progenitor cells, osteoblasts, and preosteoblasts, as well as various stem/progenitor cells with multipotent or pluripotent differentiation potential (Table 4).

**Table 4:** Summary of cellular components for 3D bioprinted osteochondral scaffolds.

Cell category	Cell type	Advantages	Disadvantages	References
Tissue-specific cells	Chondrocytes, cartilage progenitor cells, osteoblasts, and preosteoblasts	1. Promising cell options for osteochondral repair 2. Restricted to chondrogenic or osteogenic lineage 3. Limited severe clinical issues 4. More sufficient than stem cells	1. Donor site morbidity 2. Dedifferentiation occurred during cellular expansion 3. Limited cells available	[15,58,[176], [177], [178]]
Stem (progenitor) Cells	Bone marrow-derived mesenchymal stem cells, adipose-derived mesenchymal stem cells, embryonic stem cells, and induced pluripotent stem cells	1. Easily and readily available from bone marrow, fat tissue, and synovial membrane, etc. 2. Higher differentiation capability and excellent expandability 3. Possessing the ability to resist cellular senescence	1. Potential risks of bringing on tumorigenesis 2. Not restricted to osteogenic and chondrogenic lineages 3. Formation of fibrocartilage rather than hyaline cartilage 4. Terminal hypertrophic differentiation along with mineralization leading to the replacement of cartilage by bone 5. Adipose-derived mesenchymal stem cells possessing limited chondrogenic potential 6. Cell quality in relation to the age and diseases of donors	[[179], [180], [181], [182], [183], [184], [185], [186]]
Co-culture of chondrocytes and stem/progenitor cells	Chondrocytes and stem/progenitor cells	1. Less quantity of chondrocytes required, and no need for large-scale collection of non load-bearing cartilage tissue 2. Avoiding dedifferentiation during in vitro expansion and reducing fibrocartilage formation 3. Supporting more hyaline cartilage regeneration	1. Not fully elucidated mechanism of coculture effectiveness 2. Unclear optimal ratio of chondrocytes and stem cells	[103,154,[187], [188], [189]]

#### 3.2.1 Chondrocytes and cartilage progenitor cells

Chondrocytes, as the primary cellular components of articular cartilage, play a crucial role in maintaining cartilage structure and function, making them an ideal choice for incorporation into bioprinted cartilage constructs. In vitro studies have demonstrated that chondrocytes can migrate and proliferate effectively within the cartilage layer of various bioprinted scaffolds. Meanwhile, they can express cartilage-specific proteins and maintain their morphology and phenotype. For instance, the viability of chondrocyte micropellets encapsulated in HA-Tyr hydrogels, as well as their ability to maintain chondrogenic differentiation, was assessed through live-dead staining, RT-PCR, and histological analysis [84]. The results indicated that chondrocytes in the bioink exhibited good viability, expressed high levels of chondrogenic markers, and maintained a chondrocyte differentiation phenotype. In vivo evaluations further confirmed that chondrocytes encapsulated in 3D bioprinted scaffolds contribute to articular cartilage regeneration and structural remodeling [58]. Moreover, the proliferation and differentiation of chondrocytes in bioprinted scaffolds are influenced not only by the biomaterials but also by structural and mechanical properties of the scaffolds, including shape, pore size, geometry, fiber orientation, and dimensionality. Meanwhile, chondrocyte encapsulation modifies hydrogel properties by interacting with its precursors, leading to a reduction in crosslink density [190].

Although chondrocyte-laden bioinks have been widely used in the fabrication of 3D bioprinted osteochondral scaffolds, several challenges persist. First, chondrocytes used in 3D bioprinting are typically isolated from healthy articular cartilage in non-weight-bearing regions and generally require 3–5 weeks of in vitro culture before incorporation into bioinks [176]. The low yield and limited number of chondrocytes, coupled with their restricted proliferative capacity, often result in secondary cartilage degeneration at the donor site. Second, the absence of suitable in vitro culture expansion protocols increases the risk of dedifferentiation into a fibroblast-like phenotype [15]. Third, with each passage, chondrocytes become larger and flatter, and by the fifth passage, they often fail to reach confluence [58]. To overcome the limitations of isolating chondrocytes from non-weight-bearing cartilage regions, researchers have explored alternative cell sources, such as chondrocytes derived from the nasal septum and auricular cartilage [191]. Furthermore, strategies such as the supplementation of bioactive molecules and the use of bioreactors have shown promise in enhancing chondrocyte proliferation and maintaining the chondrogenic phenotype during in vitro culture.

Chondral progenitor cells are located in the superficial layer of articular cartilage, comprising approximately 0.1 %–1 % of the total cartilage. These cells exhibit self-renewal capacity in vitro and retain the potential to differentiate into osteoblasts, chondrocytes, and adipocytes, even after 60 generations of expansion [192]. More importantly, chondrocytes differentiated from chondral progenitor cells maintain their chondrogenic phenotype more effectively and exhibit fewer signs of degenerative changes compared to those differentiated from MSCs [177]. For example, in the cartilage layer of a 3D bioprinted osteochondral scaffold composed of 7 % GelMA and 3 % AlgMA, encapsulated chondral progenitor cells exhibited high viability [124]. These cells survived, proliferated normally, and differentiated into chondrocytes. Additionally, the cartilage ECM, including collagen type II and aggrecan, significantly increased in the 3D culture system. Chondral progenitor cell laden hydrogel scaffolds demonstrated effective cartilage regeneration in in vivo experiments, resulting in a 23.5 % increase in newly formed cartilage compared to acellular hydrogels.

Fetal cartilage-derived chondral progenitor cells exhibit superior proliferative capacity and multipotency compared to MSCs, enabling the fabrication of osteochondral tissues within a single construct [193]. To promote the differentiation of fetal cartilage-derived progenitor cells into both cartilage and bone, Yu et al. developed a polydimethylsiloxane-based co-culture system using 3D bioprinting [194]. This system employed distinct chondrogenic and osteogenic media to induce the differentiation of fetal cartilage-derived progenitor cells into osteochondral tissues. These findings highlight the potential of chondral progenitor cells as a valuable source of seed cells for osteochondral tissue engineering. However, challenges related to their limited availability, along with safety concerns regarding potential chromosomal abnormalities following extensive passages, necessitate further investigation.

#### 3.2.2 Osteoblasts and preosteoblasts

In the construction of the subchondral bone, several cell types commonly used in bone tissue engineering have been applied in 3D bioprinting. Osteoblasts and preosteoblasts, as progenitor cells of bone, exhibit stable osteogenic differentiation phenotypes and have been extensively investigated for their potential in subchondral bone regeneration. Preosteoblasts differentiate into osteoblasts in vitro and promote bone formation in vivo [178]. Osteoblasts encapsulated within GelMA/nHAP hydrogels maintained superior bioactivity and exhibited upregulated expression of osteogenic markers after one week of bioprinting [84]. Similarly, when incorporated into alginate-MC mixed bioinks and exposed to BMP-2, osteoblasts demonstrated upregulation of osteogenic gene expression [195].

#### 3.2.3 Stem/progenitor cells

Mature chondrocytes and osteoblasts exhibit slow proliferation rates, making it challenging to meet the high demand for cell numbers in bioprinting. In contrast, multipotent stem/progenitor cells are readily available and proliferate rapidly. They can also differentiate into various tissue-forming cells, including chondrogenic and osteogenic cells for cartilage and bone regeneration, respectively. Consequently, an increasing number of studies have integrated stem cells with 3D bioprinting for the repair of osteochondral defects. The most commonly used stem cell types include MSCs, embryonic stem cells (ESCs), and induced pluripotent stem cells (iPSCs).

MSCs can be readily sourced from various tissues, including bone marrow, adipose tissue, synovium, muscle, amniotic fluid, and peripheral blood [179]. They are easily isolated, exhibit low immunogenicity, present minimal risk of teratoma formation, and are not associated with the ethical concerns typically linked to other stem cell types. MSCs demonstrate robust proliferative capacity and multipotent differentiation potential, enabling their differentiation into chondrocytes and osteoblasts under appropriate in vivo or in vitro conditions. As a result, MSCs are considered a promising cell source for osteochondral tissue repair. MSCs have been extensively documented to spread and proliferate within bioinks composed of various biomaterials [180]. In the presence of chondrogenic or osteogenic signals, MSCs encapsulated in bioinks are prone to differentiate into chondrocytes or osteoblasts, then producing cartilage or bone ECM [181,182]. Furthermore, extensive studies have shown that the therapeutic efficacy of MSCs largely depends on their ability to produce trophic factors [58]. By interacting with local biochemical and biomechanical stimuli, MSCs release a variety of growth factors that facilitate osteochondral repair.

BMSCs can be harvested in substantial quantities from various sites within the bone marrow, making them highly suitable for osteochondral tissue engineering [196]. BMSCs possess robust proliferative capacity, chondrogenic differentiation potential, and the ability to maintain their phenotype in vitro. Bioinks composed of GelMA and BMSCs enabled rapid photocrosslinking and spontaneous covalent bonding, providing a favorable microenvironment that supported high cell viability, interaction, migration, and proliferation [87]. In vivo studies demonstrated that cartilage scaffolds bioprinted with GelMA-BMSCs bioinks promoted the regeneration of cartilage collagen fibers at the defect site, showing significant therapeutic efficacy. Furthermore, a BMSC-laden 3D bioprinted multilayer scaffold composed of HAMA/PCL, incorporating kartogenin (KGN) and β-TCP, was designed to repair site-specific osteochondral defects [197]. In vitro, BMSCs within the scaffold survived, proliferated, and produced substantial amounts of cartilage-specific ECM. In an animal model, BMSC-laden scaffolds stimulated the production of type II collagen and inhibited the expression of interleukin (IL)-1β.

In addition to their chondrogenic differentiation potential, BMSCs exhibit potent osteogenic differentiation abilities. They play a pivotal role in subchondral bone regeneration and serve as catalysts for optimal adaptation for constructing subchondral bone-mimetic components within osteochondral scaffolds. When cultured in osteogenic medium, BMSC encapsulated in GelMA- AlgMA composite hydrogels demonstrated upregulated expression of bone-specific markers, with Alizarin Red staining indicating a gradual increase in osteochondral ECM [124]. Heatmap analysis revealed significant upregulation of osteogenic genes, including Gli3, Ddr2, and Bmp2/4/7. In vivo, BMSC-laden hydrogels promoted trabecular bone regeneration and mature bone formation, as evidenced by extensive areas of red-stained mineralized tissue. Furthermore, researchers employed 3D bioprinting to directly assemble lineage-induced BMSCs and matrix materials, resulting in an integrated osteochondral biphasic scaffold [146]. The bone phase was constructed using a bioink composed of osteogenically induced BMSCs, HAP powder, sodium alginate, and gelatin. The cartilage phase consisted of chondrogenically induced BMSCs combined with sodium alginate and gelatin. Histological analysis revealed seamless integration between the regenerated cartilage and subchondral bone, indicating the formation of a nearly native osteochondral structure.

ADMSCs are readily accessible, can be isolated from multiple adipose tissue sources, and exhibit strong immunomodulatory properties [12]. These cells are less invasive to obtain compared to other stem cell sources, associated with fewer postoperative complications, and show significant potential as seed cells for tissue regeneration. Encapsulation of ADMSCs with bioactive factors in an acrylate β-cyclodextrin hydrogel led to the development of a multifunctional bioink, enabling the one-step fabrication of layered scaffolds via thermally assisted extrusion-based bioprinting [126]. Within the hydrogel matrix, encapsulated ADMSCs spread and migrated freely, secreting various types of collagen in response to the sustained release of melatonin (MLT) and KGN, thereby creating a localized microenvironment conducive to osteochondral regeneration. This process facilitated the deposition of a hierarchical ECM and supported osteochondral tissue arrangement, ultimately achieving full-thickness osteochondral regeneration.

Despite the advantages of ADMSCs, variability in cell yield remains a notable challenge. Comparative studies have revealed significant differences in the differentiation potential among ADMSCs derived from various adipose tissue sources [198]. According to embryological theories, IPFP-ADMSCs exhibit chondrogenic potential similar to that of stem cells from articular cartilage, showing high expression of chondrogenic-related genes such as SRY-box transcription factor (SOX)-9, cyclooxygenase (COX)-2, and aggrecan [199]. Interestingly, during knee arthroscopic surgery, a portion of IPFP-ADMSCs is often excised using a standard electromechanical surgical blade, as their isolation is typically hindered by visual field obstruction. Therefore, investigating the use of IPFP-ADMSCs for osteochondral repair could offer a novel approach to "convert waste into valuable resources". For example, researchers have successfully used 3D bioprinting technology to construct dECM scaffolds loaded with IPFP-ADMSCs, achieving dynamic integration of seed cells, bioactive factors, and scaffold materials [136]. This approach enhanced the chondrogenic differentiation potential of the cells in vitro.

It is important to note that when ADMSCs are cultured in a chondrogenic microenvironment, they may exhibit a tendency toward hypertrophic differentiation rather than maintaining a stable chondrocyte phenotype. This tendency may limit their potential for use in articular cartilage regeneration but could be beneficial for the repair of subchondral bone and osteochondral interface tissues. Studies have shown that the inclusion of a small amount of HAP, approximately 10 % of the polymer concentration, can promote both chondrogenic and hypertrophic differentiation of ADMSCs [200]. Increasing the HAP content further enhances hypertrophic and early osteogenic differentiation. Additionally, hypoxic conditions have been reported to inhibit the hypertrophic differentiation and calcification of ADMSCs by downregulating key markers such as collagen type X alpha 1, ALP, and MMP-13.

The pronounced hypertrophic tendency of MSCs under chondrogenic stimuli has prompted the exploration of alternative stem cell sources for cartilage repair, including pluripotent ESCs and iPSCs. ESCs have garnered significant attention in cartilage and osteochondral tissue engineering due to their capacity to provide an almost unlimited supply of chondrogenic cells [183]. However, their clinical application remains limited by several challenges, including tumorigenicity, risk of disease transmission, immune rejection, and ethical concerns [184]. To circumvent the ethical issues associated with ESCs, iPSCs derived from somatic cells present a promising alternative. Owing to their robust proliferative and differentiation capacities, iPSCs hold significant potential for widespread use in regenerative medicine. Their chondrogenic and osteogenic potential has been demonstrated in both in vitro and in vivo models of osteochondral tissue repair. For instance, Ko et al. compared the chondrogenic potential of iPSCs and BMSCs, finding that chondro-induced iPSCs exhibited higher glycosaminoglycan content and more pronounced chondrocytic features than chondro-induced BMSCs [185]. Nevertheless, several challenges remain, including the development of optimized protocols for cell isolation, differentiation, and purification ex vivo, as well as the risk of tissue malformation following in vivo application [186].

#### 3.2.4 Co-culture of chondrocytes and stem/progenitor cells

Recently, the co-culture of chondrocytes and stem/progenitor cells in 3D bioprinted scaffolds has gained attention as a strategy to enhance chondrogenesis. Although chondrocytes are typically considered the preferred cell source for the cartilage layer in bioprinted scaffolds, their propensity to dedifferentiate during in vitro culture limits their practical application. In contrast, MSCs require extensive chondrogenic induction to produce cartilage, a process that may result in hypertrophy and calcification, resembling endochondral ossification. Consequently, researchers have begun exploring co-culture models that combine chondrocytes and MSCs to leverage the advantages of both cell types in cartilage regeneration.

For instance, GelMA hydrogels loaded with chondrocytes and BMSCs, serving as the cartilage layer, preserved the chondrocyte phenotype and promoted BMSC differentiation into chondrocytes through cell-cell interactions [154]. Immunofluorescence imaging and qPCR analysis revealed increased proteoglycan and type II collagen content, as well as upregulated SOX-9 expression in GelMA hydrogels. Mechanistically, several studies have demonstrated that MSCs secrete factors such as fibroblast growth factor (FGF)-1, FGF-2, TGF-β3, and insulin-like growth factor (IGF)-1, which promote chondrocyte proliferation [187,188]. Moreover, Chen et al. reported that BMSCs induced high Kindlin-2 expression in chondrocytes, subsequently activating the PI3K/Akt/mTOR signaling pathway, which further promotes cartilage regeneration [189].

**Fig. 8:**
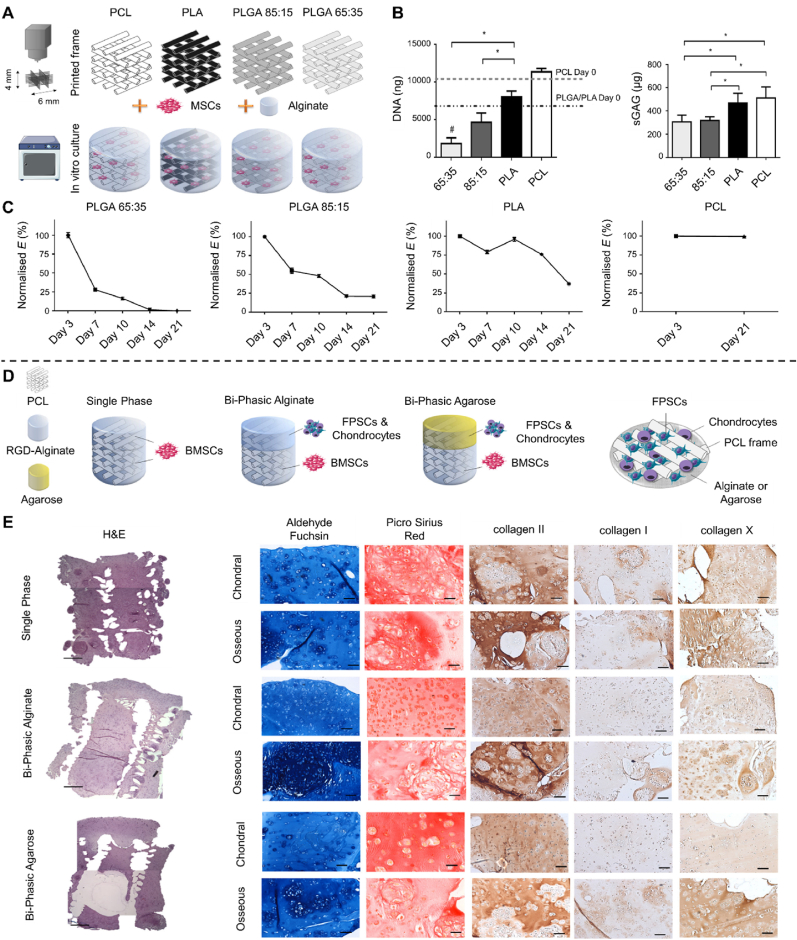
3D bioprinting of fibre-reinforced cartilaginous templates for the regeneration of osteochondral defects. (A) 3D printed polymer frames were combined with cells encapsulated in alginate. (B) Biochemical analysis at day 28 for DNA and sGAG. (C) Young's modulus normalised to day 0 of each experimental group. (D) Schematic diagram of the fabrication of bioprinted monophasic and biphasic scaffolds. (E) Staining of histological sections for H&E, Aldehyde Fuchsin, Picro Sirius Red and collagen types II, I and X. PCL: Polycaprolactone; PLA: Polylactic acid; PLGA: Poly(lactic acid-co-glycolic acid); MSCs: Mesenchymal stem cells; sGAG: Sulfated glycosaminoglycans; BMSCs: Bone marrow-derived mesenchymal stem cells; RGD:Arginine-glycine-aspartic acid; FPSCs: Infrapatellar fat pad derived stem/stromal cells. Reproduced with permission [103]. 2020, Elsevier. (For interpretation of the references to colour in this figure legend, the reader is referred to the Web version of this article.)

In another study, Critchley et al. employed IPFP-derived stem cells and chondrocytes within a PCL fiber-reinforced alginate hydrogel to bioprint a biphasic osteochondral scaffold (Fig. 8) [103]. Co-culturing these two cell types enhanced the regeneration of hyaline cartilage tissue. Although the total accumulation of sulfated glycosaminoglycans in engineered cartilage tissue from fat pad stem cells and chondrocytes did not significantly increase, a notable increase in DNA content was observed. Histological staining confirmed the presence of calcium and type I collagen in the osseous regions of the MSC-laden scaffolds and alginate-PCL controls, whereas no such staining was observed in the cartilage layer region containing co-cultured cells.

In summary, these results suggest that the co-culture of chondrocytes and stem cells represents an effective strategy for selecting seed cells for the cartilage layer of bioprinted scaffolds. This co-culture system requires only a small number of chondrocytes to maintain a stable chondrogenic phenotype, reducing the need for large-scale cartilage harvesting from non-weightbearing areas. Besides, this co-culture system mitigates chondrocyte dedifferentiation during in vitro expansion, minimizing fibrous cartilage formation and promoting hyaline cartilage regeneration.

### 3.3 3D bioprinted signaling cues for osteochondral scaffolds

Scaffolds alone often fail to provide sufficient support for osteochondral tissue regeneration. In osteochondral defects, 3D bioprinted scaffolds loaded with chondrocytes or MSCs frequently result in the formation of fibrous cartilage rather than the desired hyaline cartilage [31]. Since fibrocartilage differs from hyaline cartilage in structure and function, the inclusion of specific signaling factors to guide and accelerate cell growth has proven beneficial [201]. Biochemical signaling factors used in osteochondral repair include naturally occurring growth factors, synthetically engineered small molecules, hormones, and genes. These factors activate specific signaling pathways, stimulate the expression of relevant proteins, and mediate the growth, proliferation, and differentiation of both endogenous and exogenous tissue forming cells [58]. Among the most widely employed growth factors in 3D bioprinted osteochondral scaffolds are TGF-β, BMPs, and platelet-rich plasma (PRP). KGN is a typical synthetic molecule, while hormones such as PTH, triiodothyronine (T3), and MLT are also commonly used. Furthermore, microRNAs are increasingly utilized as regulatory factors in bioprinted osteochondral scaffolds.

#### 3.3.1 Growth factors

TGFs are a family of polypeptides that regulate various cellular behaviors, including growth, proliferation, and differentiation [202]. They are primarily classified into two types: TGF-α and TGF-β, each with unique amino acid sequences and specific receptors. Among them, TGF-β plays a pivotal role in regulating essential cellular processes, such as self-renewal, adhesion, migration, and tissue homeostasis [203]. Importantly, TGF-β induces chondrogenic differentiation of MSCs and stimulates the synthesis of cartilage-specific ECM components. It also reduces the activity levels of catabolic factors, such as IL-1 and MMPs. Therefore, TGF-β is one of the most commonly utilized growth factors to promote cartilage regeneration in 3D bioprinted osteochondral scaffolds [204]. TGF-β1 and TGF-β3, in particular, have demonstrated beneficial therapeutic effects across various bioinks [9]. However, the expression of TGF-β1 and TGF-β3, as well as their receptors, exhibit temporal and spatial variations across different zones of the osteochondral unit and during different growth phases [205]. Studies have shown that, compared to the moderate chondrogenic effects of TGF-β3 on MSCs, TGF-β1 significantly upregulates the expression of cartilage-related genes [202]. TGF-β1 also enhances the expression of cell adhesion molecules and promotes cell aggregation, whereas TGF-β3 primarily facilitates cell proliferation.

A bioink composed of dECM and silk fibroin was developed to bioprint a bilayered scaffold [138]. Designed to encapsulate TGF-β1 and BMP-2, the scaffold functions as a controlled release system to promote the directed differentiation of BMSCs. In vivo experiments demonstrated that scaffolds loaded with these growth factors effectively promoted osteochondral regeneration in a rabbit knee joint model. Additionally, a study by Kilian et al. employed extrusion-based core-shell bioprinting as an effective technique for spatially controlled delivery of various cell types and differentiation factors within distinct compartments of hydrogel strands [195]. In this system, chondrocytes and osteoblasts were incorporated into the shell, while TGF-β3 and BMP-2 were delivered by a Laponite-supported core, which served as a central depot for these factors. This configuration facilitated the directed differentiation of cells located near the core. The effectiveness of this system in locally stimulating cell differentiation was confirmed by gene expression analysis. Results showed chondrogenesis in primary chondrocytes and, to a lesser extent, osteogenesis in primary osteoblasts.

BMPs are a group of highly conserved functional proteins with similar structures, belonging to the TGF-β superfamily. Initially identified in the 1960s for their ability to induce ectopic bone formation, BMPs have since been recognized for their broader roles beyond the skeletal system [206]. These proteins are essential regulators of diverse cellular processes and serve as key morphogens during embryonic development and in the maintenance of tissue homeostasis [207]. In osteochondral tissue, BMPs are particularly noted for their ability to induce ectopic bone and cartilage formation, a process that mimics embryonic endochondral ossification [58]. To date, approximately 20 members of the BMP family have been identified, including BMP-1 to BMP-18, BMP-3b, and BMP-8b. Among them, BMP-1, BMP-5, BMP-9, BMP-13, and BMP-14 have been shown to induce chondrogenic differentiation and cartilage formation in MSCs. BMP-3, BMP-4, and BMP-8 primarily promote osteogenic differentiation. In addition, BMP-2 and BMP-7 are particularly important for their dual roles in osteogenic and chondrogenic differentiation, making them the most widely used BMPs in osteochondral tissue engineering [58].

A study comparing the therapeutic effects of IGF-1 and BMP-2 loaded into gradient scaffolds for osteochondral defect repair revealed that BMP-2 promoted bone formation in the early stages, whereas IGF-1 supported cartilage regeneration in the later stages [208]. Additionally, BMP-2 has been shown to promote the formation of vascular networks at osteogenic sites, further supporting subchondral bone regeneration [145]. Several studies have confirmed the efficacy of BMP-2 in promoting osteogenic differentiation and osteogenesis in both MSC-laden and cell-free 3D bioprinted scaffolds for the treatment of osteochondral defects, both in vitro and in vivo [138,195]. For example, Abaci et al. employed multi-material bioprinting to fabricate biphasic HAMA-based support hydrogels with spatially controlled distributions of polymer concentration, TCP particles, and BMP-2 [86]. When the polymer concentration was maintained at a constant level, the spatial release of BMP-2 resulted in MSC strands exhibiting significantly enhanced cellular spreading and increased aspect ratios, leading to a marked upregulation of local osteogenesis. Further studies are needed to elucidate the precise regulatory mechanisms by which BMPs influence chondrogenesis and osteogenesis in MSCs under different biochemical and biomechanical conditions.

PRP is an autologous blood derivative containing a variety of cytokines and other protein components. The platelet concentration in PRP is typically five times that of physiological plasma levels (100-300 × 10^9^ platelets/L) [209]. Upon activation, platelets release a broad spectrum of growth factors, including platelet-derived growth factor (PDGF), TGF-β, vascular endothelial growth factor (VEGF), epidermal growth factor (EGF), IGF-1, and FGF [209]. Therefore, PRP is frequently used as a source of growth factors and chemokines in osteochondral tissue engineering. These factors collectively promote the migration, proliferation, and differentiation of chondrocytes and BMSCs, recruit therapeutic cells, and accelerate ECM synthesis [210]. However, due to the unique biological properties of PRP, in which growth factors are released in a cascade upon platelet activation, PRP does not persist long-term in vivo. To overcome this limitation, recent strategies have focused on encapsulating PRP within bioinks for use in 3D bioprinting. This approach allows the fabrication of osteochondral scaffolds with sustained release profiles, thereby prolonging the activity and enhancing the efficacy of PRP-mediated tissue regeneration.

Irmak et al. developed a novel system combining microwave-induced GelMA/PRP bioinks with periodic polychromatic light exposure [211]. This approach enabled sustained and controlled release of growth factors, preserving PRP bioactivity while maintaining favorable mechanical properties. In vitro studies demonstrated that the proliferation and differentiation of ATDC5 cells were enhanced in the GelMA/PRP hydrogel with periodic light exposure, without the need for external chemical agents. Furthermore, the PRP-GelMA hydrogel scaffold not only directly promoted the migration, proliferation, and osteogenic and chondrogenic differentiation of BMSCs, but also modulated the polarization of immune cells into subtypes favorable for tissue repair [98]. As the repair process progressed, the PRP-GelMA scaffold treatment group exhibited a gradual reduction in the proportion of M1 macrophages, while M2 macrophages transitioned from the M2a to the M2c subtype. These processes synergistically facilitated tissue repair, resulting in a stable therapeutic effect over an extended period.

#### 3.3.2 Synthetic molecule

KGN, a nonprotein chondro-inductive agent, was identified from over 22,000 structurally diverse and heterocyclic drug-like molecules [212]. KGN has attracted considerable attention for its potent chondroprotective effects, demonstrating the ability to preserve chondrocyte viability under osteoarthritic conditions in both in vitro and in vivo models [212]. Beyond its protective effects, KGN promotes MSC chondrogenic differentiation in a dose-dependent manner, primarily by activating the Smad4/Smad5 signaling axis and modulating the CBFβ-RUNX1 transcriptional pathway [213]. Notably, KGN also induces the homing of endogenous MSCs to sites of cartilage injury, thereby promoting cartilage repair and regeneration without the need for exogenous stem cell transplantation [214]. These unique properties position KGN as a promising small-molecule therapeutic for applications in osteochondral tissue engineering and regenerative medicine.

Unlike conventional growth factors prone to instability and inactivation, KGN remains stable during storage and transport, even at room temperature [215]. Consequently, strategies for utilizing KGN are diverse, including direct intra-articular injection, incorporation into particle or thermogel drug delivery systems, and encapsulation within cartilage and osteochondral tissue engineering scaffolds [9]. However, KGN's low water solubility and rapid clearance from the joint cavity due to continuous synovial fluid renewal present major challenges for its direct clinical application. Compared to KGN administration alone, osteochondral scaffolds fabricated via 3D bioprinting enable sustained release of KGN [216]. This approach prolongs KGN retention in the joint cavity, providing targeted and long-term therapeutic effects for osteochondral defects. For example, Liu et al. utilized host-guest chemistry to encapsulate KGN into β-cyclodextrin nanoboxes, which were then embedded into a HA-based hydrogel alongside MSCs [217]. The results indicated that KGN was released in a sustained manner through molecular detachment from β-cyclodextrin and polymer degradation, effectively supporting MSC chondrogenic differentiation and cartilage regeneration.

#### 3.3.3 Hormonal substances

PTH is an anabolic agent approved by the FDA for the treatment of osteoporosis, capable of regulating bone remodeling and calcium metabolism [218]. Primarily secreted by the parathyroid glands, PTH has been shown to prevent cartilage degeneration, inhibit chondrocyte hypertrophy, and maintain the stability of the hyaline cartilage phenotype [219]. It also promotes chondrocyte proliferation and cartilage regeneration within osteoarthritic microenvironments, contributing to the repair of both cartilage and subchondral bone at osteochondral defect sites [220]. Building on these findings, 3D bioprinting has been employed to construct a biphasic scaffold using GM + silk-PTH/GM + silk fibroin methacryloyl for comprehensive osteochondral defect repair [121]. In vitro, the GM + silk-PTH bioink suppressed chondrocyte hypertrophy and promoted the production of cartilage ECM. In vivo, the bioprinted scaffold significantly enhanced osteochondral regeneration while preserving the hyaline cartilage phenotype.

Previous studies examining the endochondral differentiation of BMSCs in vitro have explored the roles of various factors that promote hypertrophic differentiation. T3 is one such factor, shown to significantly induce hypertrophy in human BMSC pellets at a concentration of 1 nM [221]. In vitro, T3 acts directly on chondrocytes, promoting hypertrophy and terminal differentiation, giving rise to increased collagen type X alpha 1 mRNA expression and elevated ALP activity in the culture medium [222,223]. Additionally, in vivo studies have suggested that increased dosages of T3 can enhance osteogenesis and mineral deposition [224]. Chawla et al. developed a silk-gelatin-based bioink encapsulating T3 and TGF-β1-activated BMSCs, and fabricated the bone layer via 3D bioprinting [225]. A combinatorial effect resulting from the addition of T3, together with the activation of endochondral ossification pathways, stimulated the PTH, IHH, and Wnt/β-catenin pathways. This activation significantly enhanced the osteogenic differentiation potential of MSCs and promoted mineralization.

Recent studies have emphasized the role of circadian rhythm-related hormones, such as MLT, in maintaining skeletal health. First identified by Aaron and colleagues in 1958, MLT is a bioactive compound primarily synthesized by the pineal gland [226]. However, it is also produced by several other tissues, such as the retina, skin, liver, intestine, ovaries, testes, and bone marrow. MLT exerts various physiological functions, including anti-aging, antioxidant, analgesic, and sedative effects [227]. In osteochondral tissues, MLT promotes beneficial effects through its antioxidant, anti-inflammatory, and bone-preserving actions, helping to counteract bone formation suppression induced by oxidative stress [228].

**Fig. 9:**
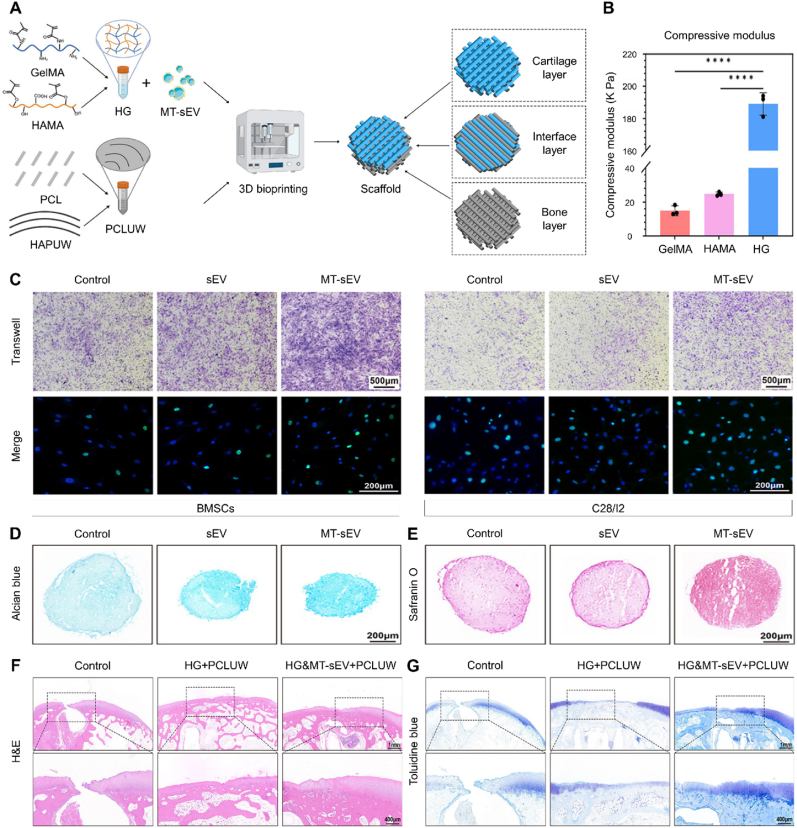
3D bioprinted biomimetic multilayer scaffolds coordinated with sleep-related small extracellular vesicles to enhance osteochondral regeneration. (A) Schematic illustration of biomimetic multiple scaffolds prepared by 3D bioprinting. (B) Compressive modulus of GelMA, HAMA, and HG hydrogels. (C) Transwell experiment and merge staining of BMSCs and C28/I2 cells to evaluate the effect of MT-sEV on cell migration and proliferation, respectively. (D) Alcian blue staining of chondrocyte spheres. (E) Safranin O staining of chondrocyte spheres. (F) H&E staining of cartilage defect repair in a rabbit model at 12 weeks. (G) Toluidine blue staining of cartilage defect repair in a rabbit model at 12 weeks. GelMA: Gelatin methacryloyl; HAMA: Methacrylated hyaluronic acid; PCL: Polycaprolactone; HAPUW: Hydroxyapatite ultralong nanowire; HG: Methacrylated hyaluronic acid and gelatin methacryloyl; MT: Melatonin; sEV: Small extracellular vesicles; MT-sEV: Sleep-related small extracellular vesicles. Reproduced with permission [113]. 2024, Wiley. (For interpretation of the references to colour in this figure legend, the reader is referred to the Web version of this article.)

It is now well-established that MLT exerts a pro-regenerative effect on cartilage through its multifaceted actions, including the reduction of inflammation, modulation of the circadian rhythm, and promotion of ECM synthesis [227]. In particular, MLT has been shown to modulate oxidative stress homeostasis, thereby promoting macrophage polarization toward the anti-inflammatory M2 phenotype [229]. This immunomodulatory effect contributes to delaying osteoarthritis progression and enhancing cartilage regeneration. In a study by Li et al., the cartilage layer of a bioprinted osteochondral scaffold was fabricated using a double-network hydrogel composed of HAMA and GelMA, loaded with MLT-related small extracellular vesicles (MLT-sEV) (Fig. 9) [113]. MLT-sEV demonstrated significant effects on cell migration, proliferation, chondrogenic differentiation, and ECM deposition. Additionally, MLT-sEV inhibited macrophage polarization toward the M1 phenotype while promoting polarization toward the M2 phenotype. This process, coupled with the regulation of inflammatory cytokine expression, enabled chondrocytes to sustain their ECM synthesis capacity, thereby facilitating cartilage regeneration.

In subchondral bone, MLT significantly enhances the osteogenic differentiation of MSCs by activating the AMPK signaling pathway [228]. The activation upregulates key transcription factors, including FOXO3a and RUNX2, which mediate cellular responses to oxidative stress and promote osteogenesis. Additionally, MLT treatment downregulates the tumor necrosis factor (TNF)-α induced expression of Smad ubiquitination-related factor 1 (Smurf1), thereby reducing Smurf1-mediated degradation of Smad proteins [230]. These changes lead to the stabilization of BMP/Smad1 signaling, thereby restoring osteoblast phenotypes damaged by TNF-α [230]. More importantly, the addition of MLT promotes angiogenesis, thereby accelerating the regeneration of vascularized bone [231]. Leveraging these properties, MLT has been incorporated into bioprinted osteochondral scaffolds as a biochemical cue to promote subchondral bone repair. A recent study developed a host-guest-modulated dynamic hydrogel for 3D bioprinting of a heterogeneous, cell-laden constructs designed for osteochondral regeneration [126]. This hydrogel enabled the sustained release of encapsulated drugs via the β-cyclodextrin cavity. The construct was spatially organized with KGN incorporated in the upper region and MLT in the lower region. With promising results in both in vitro and in vivo studies, this bioink holds great potential for 3D bioprinting applications in tissue engineering.

#### 3.3.4 MicroRNAs

MicroRNAs are a class of non-coding RNAs, typically ranging from 20 to 23 nucleotides in length, encoded by the genome. These small RNA molecules regulate mRNA degradation and inhibit translation by guiding the RNA-induced silencing complex to target gene mRNA base pairs [232]. As small-molecule inhibitors of endogenous gene expression, microRNAs are pivotal in modulating a variety of cellular processes, including proliferation, differentiation, apoptosis, and disease progression. By incorporating microRNAs into bioinks, the biological behavior of stem cells can be directed towards desired outcomes, thereby improving the therapeutic efficacy of bioprinted scaffolds for osteochondral defect repair. Several microRNAs, such as microRNA-410, microRNA-140, microRNA-30a, microRNA-488, and microRNA-21, play significant roles in the chondrogenic differentiation of stem cells and may serve as potential biomarkers for regulating this process [233].

MicroRNA-410 has been demonstrated to negatively regulate its target gene Wnt3a, thereby inhibiting the Wnt/β-catenin signaling pathway during chondrogenic differentiation [234]. This suppression reduces the nuclear translocation of β-catenin and subsequently downregulates the expression of downstream targets such as b-FGF, IGF-1, and TGF-β1. These downstream effects collectively promote the chondrogenic differentiation of stem cells, particularly under TGF-β3 stimulation. In a recent study, a GelMA-MSC bioink with upregulated expression of microRNA-410 was successfully developed in vitro, and a extrusion-based bioprinted scaffold was fabricated for the repair of distal femoral condyle cartilage defects in rabbits [87]. The results demonstrated that the upregulation of microRNA-410 influenced the biological behavior of MSCs, promoting their migration, proliferation, and differentiation. The scaffold facilitated the regeneration of both the cartilage surface and the underlying subchondral tissue in the rabbit defect model.

MicroRNA-140 facilitates chondrogenesis by upregulating SOX-9 and aggrecan expression via direct targeting of RAS-like proto-oncogene A [235]. Its expression has been shown to regulate chondrogenic differentiation in cartilaginous tissues in murine models [236]. MicroRNA-21 is another extensively studied microRNA and has been shown to promote chondrocyte proliferation and ECM synthesis in cartilage [237]. Building on these findings, a 3D heterotypic scaffold was bioprinted using spheroids of ADMSCs transfected with microRNA-148b for the osteogenic layer, and a combination of microRNA-140 and microRNA-21 for the chondrogenic layer [238]. Results demonstrated that co-delivery of microRNA-140 and microRNA-21 promoted chondrogenic differentiation of ADMSC spheroids, while microRNA-148b induced osteogenesis. Furthermore, the bioprinted construct exhibited distinct osteogenic and chondrogenic layers, retained structural integrity, and enhanced cell proliferation. In conclusion, microRNA mimic-based regulation of stem cell differentiation holds great promise for advancing osteochondral tissue engineering.

## 4 Architectures of gradient scaffolds mimicing osteochondral heterogeneity

**Fig. 10:**
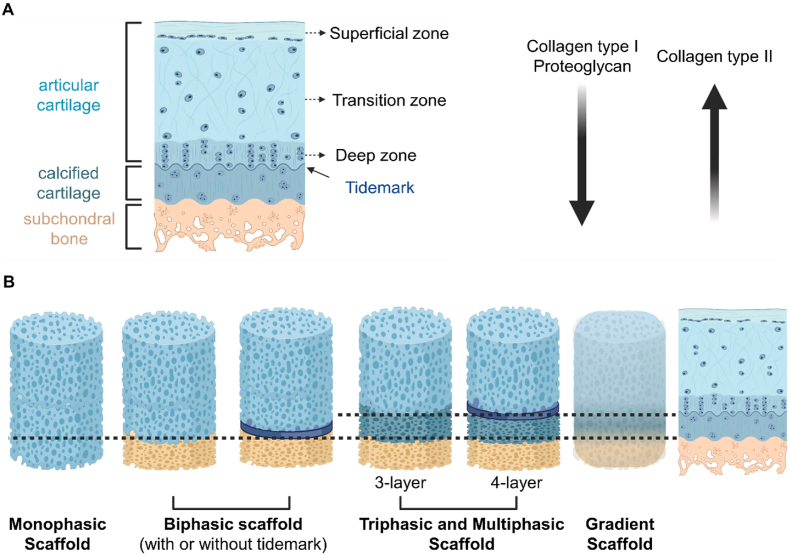
Gradient structure of osteochondral tissue and main design strategies for gradient osteochondral scaffolds. (A) Schematic illustration of gradient structure of osteochondral tissue. (B) Schematic representations of different architectural design of gradient osteochondral scaffolds. Reproduced with permission [57]. 2023, Elsevier.

Osteochondral tissue exhibits a highly organized hierarchical structure, which contributes to its distinct biological properties and structural complexity. To effectively regenerate osteochondral defects, an ideal scaffold should not only consist of high-performance bioinks but also structurally resemble the natural osteochondral unit, simulating its intricate gradient variations. Currently, the design of osteochondral scaffolds supporting both cartilage and subchondral bone regeneration can be broadly categorized into two types: multilayer scaffolds and gradient scaffolds (Fig. 10). Multilayer scaffolds are further classified into monophasic, biphasic, triphasic, and multiphasic scaffolds, each consisting of distinct but integrated layers. The material composition and scaffold geometry may vary according to the specific requirements of the tissue being regenerated. In contrast, gradient scaffolds are typically fabricated from homogeneous materials but differentiated into continuous layers through varying pore sizes or the inclusion of other components, such as mineral particles, growth factors, or cells [57,239]. Over the past decades, osteochondral scaffolds in tissue engineering have evolved from simple monophasic scaffolds to more complex biphasic, triphasic, multiphasic, and gradient scaffolds, which more effectively mimic the heterogeneity of natural osteochondral tissue [9].

Various techniques have been developed to integrate multiphasic and continuous gradients into the fabrication of osteochondral scaffolds. These techniques include 3D printing, microfluidic-based methods, electrospinning, light-mediated hydrogel formation, centrifugation, magnetic field control, and buoyancy-driven approaches [9]. Among these, additive manufacturing technologies, particularly 3D printing and 3D bioprinting, are the most established and widely applied methods for constructing scaffold gradients. The layer-by-layer assembly characteristic of 3D bioprinting enables precise spatial arrangement and distribution of biomaterials, seed cells, and bioactive factors [240]. This ability facilitates the creation of osteochondral scaffolds with tissue-specific layered or gradient structures. Therefore, 3D bioprinting offers significant advantages in constructing biomimetic osteochondral scaffolds. This section primarily discusses the cutting-edge advancements and specific applications of 3D bioprinting in developing scaffold gradients.

### 4.1 Monophasic scaffolds

Monophasic scaffolds are the earliest type of layered osteochondral scaffolds, characterized by uniform composition, structure, and mechanical properties [201]. Biologically, monophasic scaffolds typically incorporate a single type of cell and exhibit a homogeneous microenvironment with no spatial variation [240]. Although compositionally uniform, variations in material selection and structural design (such as macroscopic shape, pore size, and pore topology) can influence the efficacy of tissue restoration [9]. The development of monophasic scaffolds primarily focuses on advancing biomaterials and optimizing fabrication processes. With the advent of 3D bioprinting, a diverse range of materials can be integrated into bioinks, allowing precise control over scaffold shape and internal architecture. This technology enables the fabrication of scaffolds with well-defined structures, enhanced mechanical properties, and improved biocompatibility. Despite the significant differences between monophasic scaffolds and the layered structure of natural osteochondral tissues, recent studies have demonstrated promising results in the use of 3D bioprinted monophasic osteochondral scaffolds. These scaffolds are capable of simultaneously repairing both cartilage and subchondral bone.

A novel hydrogel was created by hybridizing sodium alginate and gellan gum with inorganic thixotropic magnesium phosphate-based gel, followed by pre-crosslinking with Mg^2+^ to enhance osteochondral repair [241,242]. This hybrid hydrogel enabled the controlled release of Mg^2+^, promoting apatite deposition and inducing osteogenic and chondrogenic differentiation of BMSCs. Histological analysis indicated that bioprinted monophasic scaffolds constructed with this material significantly promoted new bone regeneration in the subchondral bone at 6 and 12 weeks post-implantation, thereby enhancing cartilage repair. Furthermore, one study reported the use of robotic-assisted in situ 3D bioprinting technology, which mixed HAMA and a PEG derivative to form bioink for in situ repair of osteochondral defects [243]. The bioink filled the defect region with the appropriate geometry, and the cartilage injury was repaired after 12 weeks. These results indicated the feasibility of the mentioned technology for clinical application.

Although studies have demonstrated that monophasic scaffolds can support the adhesion and growth of both chondrocytes and osteoblasts, these cells require distinct microenvironments. Monophasic scaffolds lack the ability to accommodate the different ECM environments needed for articular cartilage and subchondral bone. Additionally, the uniform porosity and mechanical properties of monophasic scaffolds fail to support the structural and functional variations essential for osteochondral repair. These limitations restrict the application of monophasic scaffolds in osteochondral tissue engineering.

### 4.2 Biphasic scaffolds

Chondrocytes and osteoblasts require distinct microenvironments for optimal growth. From a biomimetic perspective, biphasic osteochondral scaffolds designed to support the proliferation and differentiation of both cell types better align with physiological conditions. Bioprinted biphasic osteochondral scaffolds typically feature a dual-layer structure, consisting of a cartilage layer and a bone layer. The upper cartilage layer serves as a temporary cartilage matrix, while the lower bone layer provides structural and biomechanical properties that closely resemble those of natural subchondral bone tissue [244]. Together, these two layers contribute to osteochondral integration and repair. In terms of biomaterials, the cartilage layer usually incorporates hydrogels made from natural or synthetic polymers and dECM, offering high hydration capacity and mechanical properties similar to natural cartilage matrix. In contrast, the calcified subchondral bone layer demands biomaterials with superior mechanical properties, such as bioceramics and rigid polymers. Beyond material selection, each layer incorporates specific tissue-forming cells and biochemical factors to promote cartilage and subchondral bone formation more effectively [245]. Consequently, the design of these layers often incorporates a complex mixture of biomaterials, resident cells, and biological molecules to optimize tissue regeneration.

Based on differences in bioinks and structural design, bioprinted biphasic scaffolds can be broadly classified into three categories: (1) biphasic scaffolds with different bioinks but the same structural design (DB/SS-biphasic scaffolds), (2) biphasic scaffolds with the same bioinks but different structural designs (SB/DS-biphasic scaffolds), and (3) biphasic scaffolds with both different bioinks and structural designs (DB/DS-biphasic scaffolds) [245].

DB/SS-biphasic scaffolds have been extensively investigated in osteochondral 3D bioprinting. In a study by Yang et al., 3D bioprinting was used to fabricate an osteochondral biphasic scaffold incorporating BMSCs [146]. The cartilage layer was printed using an alginate/gelatin bioink, while the bone layer was fabricated with an alginate/gelatin/HAP gel. The scaffold was fabricated via successive natural overlays of the printed material, controlled by computer-aided design and air pressure, to repair full-thickness osteochondral defects. Histological and biomechanical analyses conducted 6 months post-transplantation revealed nearly complete restoration of the injured injured articular surfaces, including the formation of hyaline cartilage. The repaired cartilage was also firmly integrated with the subchondral bone and closely assimilated with the surrounding tissue. In another study, a bioprinted co-culture scaffold was designed, where the top cartilage layer consisted of hydrogel bioink loaded with chondrocytes, while the bottom subchondral bone layer contained Li-Mg-Si bioceramics-based bioinks loaded with MSCs [153]. This co-culture scaffold demonstrated excellent cell viability and supported the proliferation and differentiation of both chondrocytes and osteoblasts. Furthermore, the scaffolds exhibited effective repair in the rabbit full-thickness osteochondral defect model.

The preparation of SB/DS-biphasic scaffolds via 3D bioprinting has been rarely explored in osteochondral tissue applications, likely because a simple modification in scaffold structure may not fully leverage the bidirectional effects of chondrogenesis and osteogenesis [246]. Daly et al. proposed a multi-method composite 3D printing approach for fabricating osteochondral scaffolds [111]. First, a PCL scaffold was printed using fused deposition modeling, consisting of a cartilage layer and a bone layer. The internal microgrooves in the cartilage and bone layers measured 0.8 mm × 0.8 mm and 1.2 mm × 1.2 mm, respectively. Subsequently, a Pluronic and BMSCs-GelMA mixture was extruded into the microgrooves in the bone region. Following bioprinting, the GelMA was crosslinked using ultraviolet light, while the Pluronic was removed at 4 °C, creating spaces that facilitated material exchange between the cells and the culture medium. Finally, in the cartilage region, a BMSC suspension was printed into the microgrooves using inkjet-based bioprinting. The results demonstrated that BMSCs proliferated well after bioprinting, with cells in the microgrooves fusing with the surrounding cells by extending across the walls of the microgrooves after 10 weeks of culture. The content of glycosaminoglycans, collagen, and other substances produced by the cells was comparable to that found in human osteochondral tissue. Beside, the strain equilibrium modulus of the bioprinted cartilage closely matched that of human articular cartilage.

A series of DB/DS-biphasic scaffolds have been developed by incorporating chondrogenic and osteogenic components into the upper and lower phases of SB/DS-biphasic scaffolds. Zhang et al. fabricated a 3D bioprinted bilayered scaffolds [138]. Initially, PCL was extruded to form the framework for the bone layer, followed by the bioprinting of DBM bioink to fill the designated space. DCM bioink was then used to bioprint the cartilage layer atop the bone layer. Finally, TGF-β1 and BMP-2 were incorporated into the scaffold to promote osteochondral regeneration.

A widely used strategy for fabricating biphasic osteochondral scaffolds involves separately constructing the cartilage and bone layers, followed by their integration using techniques such as adhesion or the creation of an interface layer. This approach allows for straightforward fabrication, offering a wider range of material options and morphologies for both the cartilage and bone layers, potentially better addressing regenerative needs. However, in this method, the cartilage and bone layers in the resulting scaffolds remain largely independent, lacking a well-defined intermediate phase. This issue can also arise when a separately fabricated ceramic or 3D printed scaffold is used for the bone phase, with the cartilage phase typically composed of different biological components or cells. In contrast, while material selection for 3D bioprinting is somewhat limited, it allows for the deposition of connected layers, facilitating superior integration of the cartilage and bone phases within bilayer scaffolds. Several studies have explored methods to enhance the integration of these phases during the 3D bioprinting process.

For instance, a biphasic multicellular 3D bioprinted scaffold with a dual-layered structure was designed [154]. The upper cartilage layer incorporated chondrocytes and BMSCs in GelMA, while the lower subchondral bone layer contained BMSCs in GelMA/Sr-CSH. GelMA, used as the matrix material in both layers, served as the continuous phase, effectively preventing interface barriers and promoting seamless integration at the osteochondral interface. The results showed that the biphasic multicellular scaffold exhibited excellent cell viability, regulated the differentiation of chondrocytes and BMSCs in vitro, and demonstrated promising repair outcomes for full-thickness osteochondral defects in a rat model. Additionally, Choe et al. developed a biphasic osteochondral scaffold incorporating a mechanically interlocking printing pattern at the osteochondral interface [247]. This interface design has the potential to facilitate load transfer and enhance interfacial integration. Stress simulation tests revealed that the mechanical interlocking pattern of PCL/GelMA and PCL/PEGDA scaffolds redirected shear stress from the upper cartilage layer to the deeper scaffold layers.

Physiological electric fields play a crucial role in cellular activities, influencing cell migration, proliferation, and differentiation [248]. Piezoelectric materials generate electric charges under mechanical loading, a phenomenon known as the piezoelectric effect. In osteochondral tissue, piezoelectric scaffolds stimulate stem cell differentiation, chondrocyte proliferation, and type II collagen synthesis by generating local electric signals, thereby promoting tissue repair [249,250]. However, traditional piezoelectric materials often lack sufficient biodegradability, making them unsuitable for long-term implantation. Recent research proposed a bioprintable, piezoelectric-conductive scaffold composed of piezoelectric dECM and piezoelectric-conductive modified gelatin [250]. The piezoelectricity of the scaffold was achieved by modifying diphenylalanine assemblies on the pore surfaces, while its conductivity was introduced by incorporating poly(3,4-ethylenedioxythiophene). Positive charges on the upper surface of the scaffold attracted BMSCs, promoting their migration and chondrogenic differentiation, while negative charges on the lower surface induced osteogenic differentiation.

In conclusion, compared to monophasic scaffolds, biphasic osteochondral scaffolds provide distinct microenvironments that facilitate cartilage and bone regeneration, enhancing both cell-cell and cell-matrix communication [251]. However, these scaffolds lack an intermediate cartilage-bone transition layer. While 3D bioprinting theoretically enables the creation of integrated phases for bilayer scaffolds, challenges related to interface integration persist. Moreover, complications such as interface separation and scaffold fracture may occur following implantation, potentially leading to suboptimal repair outcomes [252].

### 4.3 Triphasic and multiphasic scaffolds

The incorporation of additional layers into osteochondral scaffolds has emerged as a promising strategy for restoring the complex physiological structure of osteochondral tissue. Triphasic and multiphasic scaffolds more effectively replicate the layered structure and mechanical property variations of native osteochondral tissue. A triphasic scaffold, derived from a biphasic structure, introduces an intermediate layer between the articular cartilage and subchondral bone, simulating the calcified cartilage layer of native osteochondral tissue. Calcified cartilage, a highly mineralized region located deep within the articular cartilage, connects the noncalcified cartilage to the subchondral bone. This layer has a low cell density, with few hypertrophic chondrocytes, and serves as a critical connector that withstands compressive stress within the joint [253]. It also plays a key role in dispersing lateral stress and resisting shear forces. The dense nature of calcified cartilage divides the osteochondral unit into two distinct microenvironments, limiting the exchange of interstitial fluids between the articular cartilage and subchondral bone [201]. Consequently, this structure is essential for maintaining stability in newly formed osteochondral tissue and preventing excessive subchondral bone growth into the cartilage region. Without this regulation, vascular invasion and cartilage calcification could lead to degenerative changes in articular cartilage.

Significant advancements have been made in the development of 3D bioprinted triphasic osteochondral scaffolds. The fabrication of triphasic scaffolds requires careful selection of material components, internal structural design, and evaluation of the mechanical properties of each layer [245]. Notably, the biomaterials used to construct the osteochondral interface are primarily composed of specific component ratios tailored for the fabrication of the cartilage and subchondral bone layers. For example, Li et al. employed 3D bioprinting to fabricate a biomimetic triphasic osteochondral scaffold [113]. They used MLT-sEV-loaded HAMA-gelatin methacryloyl double-network hydrogel for the cartilage layer and PCL with HAP ultralong nanowires for the bone layer, with the interface formed by alternating these two bioinks. In vitro experiments demonstrated that MLT-sEV could modulate the immune microenvironment and promote ECM secretion in each layer. In vivo experiments further validated the ability of the bioprinted triphasic scaffold to accelerate osteochondral repair.

A novel approach integrating various 3D printing technologies has been introduced to achieve secure integration at the interface between two mechanically distinct materials, specifically cell-laden hydrogels and biologically relevant ceramics and polymers [150]. To accomplish this, the researchers developed a bioceramic ink that mimicked the mineral phase of bone, specifically calcium phosphate, and employed pneumatic extrusion-based printing to fabricate subchondral bone substitutes. Subsequently, a near-field direct writing technique was used to create a polymer mesh embedded within the ceramic ink, with GelMA loaded with cells serving as the cartilage component. This interlocking design enhanced hydrogel-to-ceramic adhesion strength by more than 6.5-fold compared to non-interlocking fiber architectures, enabling greater structural stability during handling and surgical implantation in osteochondral defects ex vivo. Furthermore, the melt electrowriting meshes endowed the chondral compartment with compressive properties closely matching those of native cartilage, resulting in a 20-fold increase in strength compared to the pristine hydrogel [150]. Both the osteal and chondral compartments supported osteogenesis and cartilage matrix deposition in vitro, with the newly synthesized cartilage matrix further contributing to mechanical reinforcement at the ceramic-hydrogel interface.

**Fig. 11:**
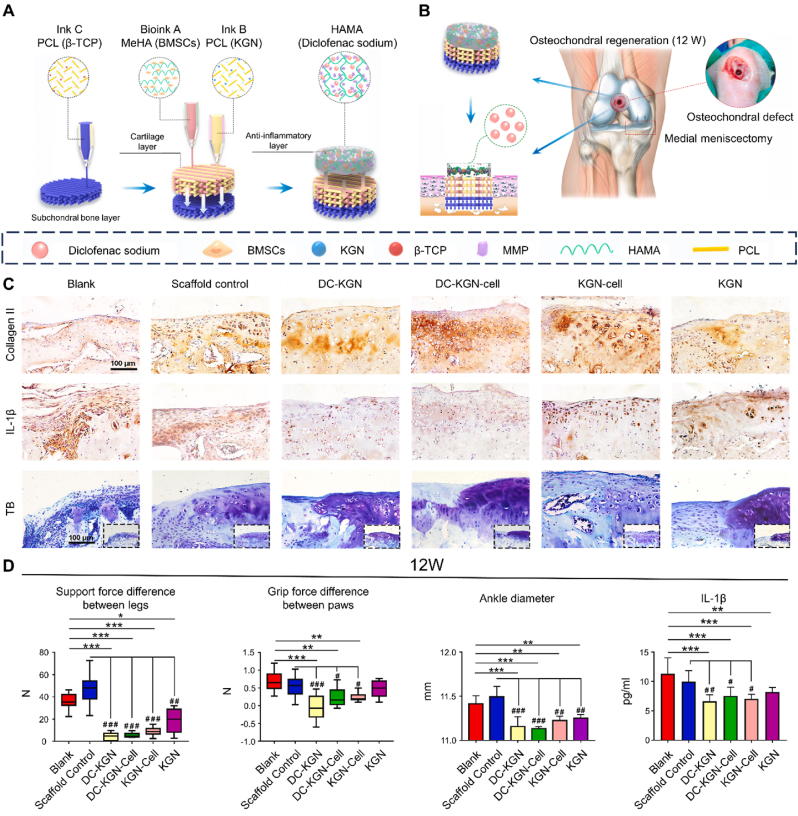
3D bioprinted triphasic scaffolds for efficient repair of osteochondral defects. (A) The fabrication of 3D bioprinted BMSC-laden biomimetic triphasic scaffolds. (B) The bioprinted scaffold was implanted into a severe joint injury rat model with osteochondral defect and medial meniscectomy for evaluation. (C) Immunohistochemical analysis of collagen II and IL-1β on the defect surface of the femur. In addition, tissue slices were stained with TB for proteoglycans. (D) The ground support force difference between two legs, the grip force difference between two paws, and the ankle diameter of the injured leg were assessed in rats at week 12 postsurgery. The serum IL-1β expression of the rats was determined by ELISA at week 12 postsurgery as well. BMSCs: Bone marrow-derived mesenchymal stem cells; KGN: Kartogenin; β-TCP: β-tricalcium phosphate; MMP: Matrix metalloproteinase; HAMA: Methacrylated hyaluronic acid; PCL: Polycaprolactone; IL-1β: Interleukin-1β. Reproduced with permission [197]. 2021, Elsevier.

In contrast to traditional bioprinted triphasic osteochondral scaffolds, Liu et al. developed a novel scaffold based on a biphasic design, employing multi-material bioprinting (Fig. 11) [197]. The 3D printed porous structure, loaded with β-TCP, mimicked the subchondral bone structure and preserved the continuous integrity of the scaffold beneath the cartilage layer, which was composed of PCL (KGN)/BMSC-laden HAMA. Additionally, a diclofenac sodium-loaded, MMP-sensitive hydrogel coating was applied to the top surface of the scaffold. This functional drug delivery system effectively managed osteoarthritis inflammation, tailored to disease progression. In vivo studies demonstrated that the BMSC-loaded biphasic scaffold exhibited superior repair efficacy in osteochondral defects. It significantly improved joint function, including parameters such as ground support force, paw grip force, and walking gait in osteoarthritis model mice.

In the development of biomimetic bioprinted triphasic osteochondral scaffolds, constructing the osteochondral interface is a critical area of research in scaffold design. This interface plays a pivotal role in maintaining joint structural integrity by preventing ectopic mineralization, bone upgrowth, and vascular invasion from the underlying subchondral bone [254]. In its natural form, the osteochondral interface is primarily composed of calcified cartilage, in which hypertrophic chondrocytes expressing type X collagen are embedded within a cartilage matrix mineralized with HAP. As the final cartilage layer adjacent to the bone, calcified cartilage must preserve the functional osteochondral connection, through which compressive, tensile, and shear forces are transmitted from the viscoelastic cartilage to the rigid, mineralized subchondral bone. Therefore, replicating the calcified cartilage zone as the osteochondral interface is crucial for the success of osteochondral tissue engineering. 3D bioprinting presents an ideal technology for functionally engineering transient interface tissues, such as the calcified cartilage layer.

Several studies have focused on using bioprinting to construct functional calcified cartilage interfaces, employing strategies such as variations in mineral content, porosity, and pore size. For instance, You et al. developed a homogeneous alginate/HAP composite hydrogel, with sodium citrate as a dispersant [255]. They reported that sodium citrate facilitated uniform dispersion of HAP particles within the hydrogel, and that HAP incorporation promoted chondrocyte secretion of the calcified matrix. Compared to the control hydrogel containing only alginate, the composite hydrogel enhanced type X collagen secretion, increased ALP activity, and stimulated mineral deposition by embedded chondrocytes. Furthermore, 3D bioprinted porous scaffolds made from the alginate/HAP composite hydrogel exhibited significantly greater alizarin red-stained matrix formation after 4 weeks of subcutaneous implantation, indicating superior potential for calcified cartilage regeneration. Additionally, a combination of alginate and GelMA with β-TCP ceramic microparticles was used to create bioinks [256]. RT-qPCR and fluorescence immunocytochemistry results revealed that TCP particle inclusion enhanced the expression of key ECM proteins, such as collagen types II and X, and aggrecan, while also significantly promoting ALP overexpression, an enzyme essential for mineral nucleation.

Overall, triphasic scaffolds better replicate the structural distribution of native osteochondral tissues, aligning closely with the requirements of tissue engineered composite scaffolds. However, challenges remain in enhancing the bonding strength at the interfaces between adjacent scaffold layers. Additionally, the design and simulation of the tidemark and calcified cartilage layer are still underdeveloped [245]. Moreover, most studies on 3D bioprinted osteochondral interfaces have overlooked the tidemark, which is challenging to induce after scaffold implantation [31]. Future research should focus on optimizing composition, parameters, and material selection. In vivo and ex vivo experiments are needed to validate these improvements and enhance scaffold integration and functionality.

Multiphase scaffolds represent some of the most complex designs in osteochondral tissue engineering, typically consisting of at least four distinct layers or more. These scaffolds extend the design of triphasic structures by incorporating additional layers within the cartilage region, aiming to create a more seamless transition between different osteochondral tissue zones. However, the design and fabrication of such intricate structures remain challenging. Moreover, there is a lack of high-quality studies demonstrating the clear advantages of multiphase scaffolds over commonly used biphasic, triphasic, and continuous gradient scaffolds.

### 4.4 Continuous gradient biomimetic scaffolds

The native osteochondral unit exhibits a complex gradient heterogeneity rather than distinct, well defined layers. To replicate this natural pattern for application in osteochondral tissue engineering, researchers have developed continuous gradient biomimetic osteochondral scaffolds. These scaffolds are designed to establish a gradual transition across the entire structure or within a localized area. Specifically, the gradients can be characterized by variations in both the chemical composition and structural characteristics. Chemical compositional gradients involve changes in the materials used and the bioactive molecules encapsulated within the scaffold. Structural gradients, on the other hand, include variations in porosity, pore size, and pore geometry. Such gradients are widely recognized as essential environmental cues that influence cell migration, differentiation, and tissue growth within the scaffold.

In the treatment of osteochondral defects, gradient scaffolds consistently outperform monophasic and biphasic layered scaffolds [32]. Unlike distinct layered structures, continuous gradient scaffolds feature smooth transitional gradients between layers, which significantly reduce the incidence of complications such as scaffold delamination. These scaffolds gradually transition in terms of composition, structure, and mechanical properties across layers, thereby enhancing overall stability through integrated design [257]. Furthermore, the continuous nature of gradient scaffolds facilitates interlayer communication between cells, promoting cellular interactions and tissue integration. The incorporation of therapeutic factors further enhances repair efficiency, ultimately enabling the in vivo integration of the implant with native tissue [57].

Various technologies have been developed to integrate gradients into osteochondral scaffolds, broadly categorized into traditional techniques, emerging technologies, and 3D printing [245]. Conventional methods, such as solvent casting, gas molding, and freeze-drying, are cost-effective and are primarily used to regulate the microstructural gradients of scaffolds to a certain extent [258]. Emerging technologies, including buoyancy, magnetic attraction, and electrical attraction, are often combined with specific materials to control the distribution of scaffold components [9,259,260]. While these two approaches offer flexibility in constructing macroscopic scaffold gradients, they face challenges in achieving precise control over microscopic structures. In contrast, 3D bioprinting enables the fabrication of continuous gradient scaffolds with high precision by regulating both macroscopic and microscopic characteristics [32]. This section summarizes the latest advancements in the application of 3D bioprinting for constructing continuous gradient osteochondral scaffolds.

The creation of chemical composition gradients is a common strategy in fabricating bioprinted continuous gradient scaffolds to replicate the native characteristics of osteochondral structures. Bedell et al. investigated the capacity of ceramic-laden bone-like and glycosaminoglycan-laden cartilage-like bioinks promote tissue-specific matrix deposition by human MSCs within multi-material scaffolds [261]. Using extrusion-based bioprinting, they fabricated two scaffold structures with varying transition regions and degrees of mixing between bone-like and cartilage-like bioinks. The gradient fiber architecture group exhibited a significant increase in chondral integration over time, with a peak push-out force of 18.5 ± 0.7 kPa on Day 21, compared to 9.6 ± 1.6 kPa on Day 1. In contrast, the segmented fiber architecture group did not exhibit a similar increase. This finding was correlated with enhanced deposition of sulfated glycosaminoglycan, observed exclusively in the gradient fiber group. Furthermore, staining results revealed enhanced cellularity and tissue-specific matrix deposition at the interface between fibers and defects in the gradient fiber group.

Recently, a multimaterial deposition system based on a microfluidic platform with a mixing compartment has been proposed, demonstrating its feasibility for depositing continuous gradients of cells and materials within 3D structures with high shape fidelity and open porosity [262]. Based on this, researchers developed bioinks that replicated the composition of the various zones found in native articular cartilage. Specifically, an alginate, GelMA, and chondroitin sulfate hydrogel, loaded with human chondrocytes and MSCs, was used to represent the cartilage phase, with its content gradually decreasing from top to bottom. The bone phase, composed of TCP microparticles and HAMA, exhibited a reverse gradient, with content decreasing from bottom to top, aiming to promote heterogeneous differentiation of stem cells within the scaffold. After 21 days of in vitro culture, the researchers observed the production of zone-specific matrices. Histological analysis of surgically induced osteochondral defects in rat trochlea further revealed the beneficial effects of the bioprinted scaffolds on tissue regeneration, compared to the untreated control group.

In designing structural gradients for osteochondral scaffolds, factors such as pore size, arrangement, and geometry are crucial, as they significantly influence osteochondral regeneration. Smaller pore sizes (125–250 μm) have been shown to enhance chondrogenic differentiation of BMSCs, thereby promoting cartilage formation [263]. In contrast, pore sizes larger than 300 μm facilitate direct osteogenesis and vascularization. Therefore, in scaffolds with a pore gradient, regions with larger pores can support vascularization and osteogenesis, while regions with smaller pores promote cartilage formation. The pore arrangement is critical for regulating cell infiltration, tissue formation, and the scaffold's mechanical properties. Studies have shown that scaffolds with longitudinally oriented pores act as channels, guiding the migration of BMSCs to the damaged cartilage regions [264]. This arrangement not only aids in cell infiltration but also ensures a more uniform distribution of cells, minimizing aggregation at the scaffold edges [264].

Golebiowska et al. developed osteochondral scaffolds with gradient structural designs to facilitate seamless transitions between tissue zones, more accurately replicating the bone-cartilage interface [110]. These multi-zonal scaffolds featured tunable porosity and pore sizes along their length. Additionally, the authors introduced a novel bioprinting technique to selectively place cells into the desired scaffold zones by concurrently printing a cell-laden hydrogel within the porous template. Live-dead staining results revealed uniform cell distribution and high cell viability in the printed cell-laden hydrogel within the osteochondral scaffolds. Similarly, Nowicki et al. used fused deposition modeling-based 3D bioprinting to fabricate a complex osteochondral scaffold with gradient pore distribution and tunable nHA incorporation, which regulated both chondrogenic and osteogenic responses [265]. Their results indicated enhanced biological and mechanical properties in scaffolds with anisotropic pore distributions, particularly compared to homogeneous, isotropic, or non-porous scaffolds. Differentiation assays indicated successful osteogenic and chondrogenic manipulation within the engineered scaffolds. Regarding pore geometry, Martinez-Moreno et al. showed that pore shape significantly influences the growth and adhesion of infrapatellar MSCs [266]. Using bioprinting, they fabricated hexagonal, square, and triangular pores, with triangular pores offering the most favorable conditions for MSCs in cartilage regeneration.

Stiffness is another critical parameter that can be precisely regulated in the fabrication of 3D bioprinted osteochondral continuous gradient scaffolds. When the biomechanical properties of the scaffold closely match those of native osteochondral tissue, stress concentrations at the interface due to mechanical mismatches are minimized, thereby improving integration between the implant and native tissue [9]. Additionally, MSCs exhibit heightened sensitivity to mechanical stimuli, responding in real time to both passive factors, such as stiffness, and dynamic stimuli, such as mechanical loading and hydrostatic pressure [267]. These stimuli are transduced into biological signals via molecules such as integrins and focal adhesion protein complexes, which subsequently regulate cellular behaviors. Consequently, many studies have focused on modulating MSC differentiation by altering the stiffness of the ECM. The stiffness gradient within scaffolds can be achieved by adjusting the concentration or composition of materials or components.

A novel bioprinting platform has been developed to enhance the chondrogenic and osteogenic differentiation of MSCs by modulating the stiffness of GelMA microgels and the volume of the matrix-filling phase [268]. Building upon this strategy, the system was further optimized by incorporating a rapidly solidifying calcium phosphate cement ("bone ink") into the cell suspension, allowing the integration of both soft and hard materials. Cells near the newly formed HAP phase underwent osteogenesis, while cells in the surrounding medium exhibited chondrogenesis. The method facilitated the formation of bone-like structures with a layered architecture within a cell-laden, gradient soft matrix, effectively generating a multiphasic osteochondral construct. This platform provides a versatile one-step biomanufacturing method, eliminating the need for stringent post-processing and advancing osteochondral disease modeling and tissue engineering.

Continuous gradient scaffolds fabricated through 3D bioprinting have successfully replicated both chemical compositions and structural characteristics, as outlined in this section. However, research on 3D bioprinted gradient scaffolds that mimic osteochondral heterogeneities remains limited. Development is still in its early stages, particularly in replicating anatomical, biological, physicochemical, and mechanical properties. Additionally, the fabrication process of gradient scaffolds is relatively complex, requiring meticulous control to ensure consistency throughout the manufacturing stages. Besides, the mechanical properties of hydrogel-based gradient scaffolds may not be sufficient to support immediate load-bearing following implantation.

## 5 Conclusion and future directions

Recent advancements in 3D bioprinting have significantly progressed osteochondral repair. Nevertheless, major technical bottlenecks and scientific challenges remain unresolved. Primarily, the osteochondral unit possesses a multiscale hierarchical structure ranging from nanometer to micrometer scales. Conventional 3D bioprinting techniques, including extrusion- and inkjet-based methods, typically offer limited resolution (10–50 μm), impeding accurate reproduction of the intricate architecture of the osteochondral unit. In addition, stereolithography-based bioprinting employs ultraviolet-triggered photopolymerization, potentially compromising cell viability and diminishing the bioactivity of incorporated signaling factors. Therefore, there is an urgent need to develop next-generation bioprinting technologies featuring higher resolution and milder processing conditions. In response, recent studies have introduced advanced bioprinting approaches, including digital light processing (DLP) bioprinting, volumetric bioprinting, and continuous chaotic bioprinting.

DLP bioprinting provides rapid printing speeds (approximately 30 mm^3^/s) and high-resolution capabilities down to about 1 μm. These attributes make it particularly advantageous for fabricating intricate cubic structures. Meanwhile, DLP bioprinting relies on localized photopolymerization of liquid monomers or oligomers activated by ultraviolet light and photoinitiators, offering significant practical benefits. Volumetric bioprinting, an advanced method employing volume slicing and light intensity compensation algorithms derived from computed tomography (CT), further improves precision. By projecting sequential 2D slices of a 3D model into a rotating print chamber, volumetric bioprinting enables simultaneous formation of bioinks and cells into 3D constructs rapidly (often within seconds). Besides, volumetric bioprinting operates under mild conditions, allowing printing with visible light at room temperature. The system maintains non-contact with the printed scaffolds, contributing to a higher cell survival rate (up to 95 %) in volumetric bioprinting applications. Moreover, continuous chaotic printing, a novel extrusion method utilizing Kenics static mixer printheads, mixes bioinks and extrudes well-defined internal lamellae, significantly increasing the interface area between adjacent bioinks. These innovative bioprinting techniques present considerable potential for simulating osteochondral units.

Secondly, osteochondral repair is an extraordinarily complex and dynamic process. The injury microenvironment undergoes multistage changes, including immune cell chemotactic infiltration, MSCs differentiation, immune factors release, growth factor secretion, and the transition from disordered to organized ECM. Therefore, the focus of osteochondral tissue engineering should be to replicate dynamic cellular behaviors, spatiotemporal cytokine release, and cell-matrix signaling interactions to regulate tissue repair. However, scaffolds fabricated via conventional bioprinting technologies remain static post-manufacturing and fail to meet these requirements. To address this limitation, four-dimensional (4D) bioprinting has emerged as a promising solution. By incorporating time as a fourth dimension, 4D bioprinting enables the fabrication of dynamic, bioactive 3D constructs. These constructs possess stimulus-responsive properties that replicate the intrinsic dynamics of native osteochondral tissue, advancing biomedical engineering applications.

Currently, 4D bioprinting remains in its early stages, as well as the availability of intelligent bioinks for osteochondral defect repair is highly limited. As a result, the design and development of intelligent bioinks represent a major challenge in osteochondral 4D bioprinting. Ideal intelligent bioinks not only exhibit printability, biocompatibility, biodegradability, and adequate mechanical properties but also demonstrate responsiveness to stimuli. Specifically, these inks are designed to react to physiological conditions or external stimuli, including physical (temperature, ultrasound, light, magnetic and electric fields), chemical (pH, humidity, biomolecules), and biological (glucose, enzymes) cues. This property enhances control over the bioprinting process and the structural and functional properties of bioprinted constructs. Additionally, these inks should be engineered to degrade at controlled rates as well, allowing for gradual replacement by native osteochondral tissue. However, current intelligent bioinks with adequate deformability and biocompatibility are largely limited to specific natural hydrogels. Moreover, most existing intelligent bioinks respond to only one or two triggers, which may be insufficient to address the complex microenvironment of osteochondral defects. Although research remains limited, the integration of intelligent bioinks with 3D bioprinting, referred to as 4D bioprinting, offers a promising transition from "static structural fabrication" to "dynamic living system engineering" for osteochondral regeneration.

Lastly, in strategies involving bioprinted scaffolds combined with bioactive factors, it is essential to rigorously validate and optimize both the dosage and release profiles of these factors. Exogenous bioactive factors typically have short half-lives and tend to degrade rapidly in vivo. Additionally, localizing the presentation of signaling molecules presents a significant challenge due to the diffusive transport properties of bioinks. As a result, effectively harnessing the biological effects of bioactive factors to specific regions remains a critical challenge. Gene therapy has emerged as a transformative technology, attracting significant attention across various fields. When integrated with bioprinted scaffolds, while ensuring safety as a fundamental prerequisite, it offers an innovative alternative to exogenous factors for promoting osteochondral regeneration.

Several methods are available for integrating gene therapy with biofabrication techniques. One approach involves encapsulating the nucleic acid and its delivery mechanism (chemical, physical, or viral) within the bioprinted scaffold during fabrication, creating a gene-activated bioink. Besides, genetic materials can be introduced into target cells by applying mechanical stress during the bioprinting process. A two-step approach is also feasible, where nucleic acids and their delivery systems are incorporated into the scaffold post-fabrication, either through biomaterial-based chemical interactions or direct loading, thereby enhancing cellular uptake. In summary, the integration of gene therapy with bioprinting not only overcomes the rapid degradation associated with exogenous biological factors but also allows for precise spatial control of distinct cell populations. Moreover, gene therapy facilitates the replication of the spatial distribution of growth and transcription factors present in natural osteochondral tissue, marking a impactful advancement in osteochondral tissue engineering.

In conclusion, 3D bioprinted osteochondral scaffolds represent a promising strategy for the repair of osteochondral defects. However, further research is essential to facilitate their clinical translation. Future studies should focus on the following key areas: (1) Advancing bioprinting technologies to achieve higher resolution while minimizing cellular damage; (2) Optimizing the dynamic responsiveness of smart bioinks to enable precise 4D regulation; (3) Integrating gene therapies with bioprinting to achieve spatiotemporal control over factor release. Advancements in 3D bioprinting technologies, bioinks, and innovative design concepts provide new opportunities for the development of biomimetic osteochondral scaffolds and the treatment of osteochondral defects.

## CRediT authorship contribution statement

**Jialin Lu:** Writing – original draft. **Yu Gao:** Writing – review & editing, Data curation. **Chen Cao:** Validation, Methodology. **Hang Wang:** Validation, Data curation. **Yaokuan Ruan:** Supervision, Conceptualization. **Keyi Qin:** Visualization, Software. **Hengyu Liu:** Software, Methodology. **Yanbo Wang:** Validation, Conceptualization. **Pengju Yang:** Software. **Yi Liu:** Conceptualization. **Yingxue Ma:** Visualization. **Zhifei Yu:** Methodology. **Yinan Wang:** Validation, Methodology, Conceptualization. **Zhuan Zhong:** Validation, Methodology, Conceptualization. **Fei Chang:** Writing – review & editing, Supervision, Funding acquisition, Conceptualization.

## Declaration of competing interest

The authors declare that they have no known competing financial interests or personal relationships that could have appeared to influence the work reported in this paper.

## Data Availability

No data was used for the research described in the article.
